# Newly Synthesized Citral Derivatives Serve as Novel Inhibitor in HepG2 Cells

**DOI:** 10.1002/open.202400112

**Published:** 2024-11-26

**Authors:** Wei Gao, Xiaoju Hua, Shengliang Liao, Zhikai Xiahou, Haikuan Yang, Lifang Hu, Yunyang Chi

**Affiliations:** ^1^ Camphor Engineering Research Center of National Forestry and Grassland Administration Jiangxi Academy of Forestry Nanchang 330032 China; ^2^ Jiangxi Agricultural University Nanchang 330045 China; ^3^ Institute for Quality & Safety and Standards of Agricultural, Products Research Jiangxi Academy of Agricultural Sciences Nanchang 330299 China; ^4^ China Institute of Sport and Health Science Beijing Sport University Beijing 100091 China

**Keywords:** Citral, Derivative, HepG2 liver cancer cell, Cell apoptosis, Anti-cancer

## Abstract

2H‐pyran compound 1 synthesized from 6‐methylpyridine‐2,4‐diol and citral, has been used for Cu‐catalyzed *N*‐arylation with a range of arylboric acids to obtain arylated pyranopyridine core structure derivatives (yield up to 77 %). Among them, compound **3 h** exhibited a much better inhibitory effect on HepG2 liver cancer cells compared to citral, and the IC_50_ value was 5.3 μM following exposure with the newly synthesized derivatives (herein named **3 h** for short in this paper), which was lower than that of the cisplatin (6.5 μM). Meanwhile, the cell‐cycle arrest of HepG2 cells occurred in the S phase, and the apoptosis of HepG2 cells was significantly increased with increasing drug concentration. In addition, real‐time fluorescence quantification PCR and Western blotting experiments showed that the expression of apoptotic protein BAX was increased, while the expression of anti‐apoptotic protein BCL2 was inhibited in a dose‐dependent fashion. The results of these experiments indicated that apoptosis was promoted in HepG2 cells via apoptotic signaling pathway activated by **3 h**. Furthermore, **3 h** effectively decreased the phosphorylation levels of PI3 K, ATK and ERK, resulting in the inhibitions of MAPK/ERK and PI3 K/ATK signaling pathways. Briefly, **3 h** has been found to show inhibitory effects on the survival of HepG2 liver cancer cells and may be used as anti‐cancer drug in the future.

## Graphical abstract

Compound 3h has been found to show inhibitory effects on the survival of HepG2 liver cancer cells.The results show that 3h induces cell‐cycle arrest in the S phase. Additionally, 3h exerts a strong pro‐apoptotic effect. Specifically, compound 3h reduces the phosphorylation of PI3K, AKT, and ERK, thereby inhibiting both the MAPK/ERK and PI3K/AKT signaling pathways.

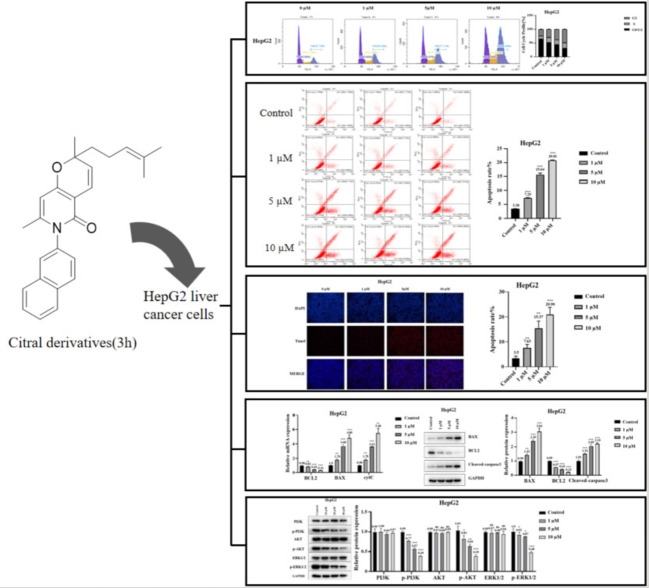

## Introduction

Hepatocellular carcinoma is the most common type of primary liver cancer, characterized by a long course, high recurrence rate, and difficult treatment.^[1]^ In recent years, various bioactive compounds present in plants, such as alkaloids, flavonoids, glycosides, saponins, tannins and terpenoids, have been found to have the potential of antioxidant and anticancer activities against liver cancer cells. They can curb the occurrence and development of liver cancer by suppressing the effects of carcinogens, improving the effectiveness of chemotherapy drugs, inhibiting the growth and metastasis of tumor cells, reducing oxidative stress, metabolic disorders, angiogenesis and chronic inflammation, promoting cell apoptosis, and some other pathways.^[2]^ In a word, it is of great significance to explore and develop the potential activities of natural compounds for the treatment of hepatocellular carcinoma.

As one of the main components of plant essential oils, citral is prone to redox reactions to produce other compounds such as geranilic acid, geraniol or nerolidol due to the relatively active chemical properties. On the other hand, citral possesses inhibitory activity in many tumor cell lines, such as breast cancer cells, cervical cancer cells, B‐lymphoma cells as well as pancreatic tumor cells.[^3^, ^4^, ^5^, ^6^, ^7^, ^8^, ^9^, ^10^] Citral not only has good anti‐tumor cell activity on its own, but also could be combined with other substances to fight against cancer. Also, as a naturally occurring monoterpenoid, it has strong antibacterial and antifungal activities and has been commonly considered to be a promising antimicrobial compound.[^11^, ^12^, ^13^] Nowadays, citral has been converted into a series of derivatives with better biological activity.[^14^, ^15^, ^16^] However, there have been few studies concerning the effects of citral derivatives on liver cancer cells.

The 2H‐pyran fundamental substructure is widespread in nature and embedded in several subclasses of natural products like pyranocoumarins and pyranoquino‐linones.[^17^, ^18^, ^19^, ^20^, ^21^, ^22^, ^23^] In addition, this oxygenated heterocycle displayed multiple reactivity profiles, such as transient non‐isolated reactive intermediates trapped in subsequent chemical transformations, and become versatile entities in synthetic organic chemistry.[^24^, ^25^] Because of the wide range of biological activities and pharmacolog‐ical properties, the 2H‐pyran heterocyclic cores were widely used in synthetic organic chemistry. Lee and coworkers developed a methodology for the synthesis of 2H‐pyran‐containing compounds by the formal [3+3] cycloaddition of 2, 4‐dihydroxyquinoline and varied *α, β*‐unsaturated aldehydes.^[26]^ It was an efficient way to make 2H‐pyranyl derivatives with a variety of substituents. In 2023, we synthesized 2H‐pyranyl derivatives from citral using Cu‐catalyzed *N*‐arylation with a wide range of arylboric acids. This N‐selective type of coupling reaction proceeds well with a broad substrate scope, providing a direct way to produce highly functional pyranoquinolinone core structure derivatives in yields of up to 80 %.^[27]^ In this study, citral was used to synthesize more stable 2H‐pyranyl derivatives for screening compounds with high activity against liver cancer cells.

## Results and Discussion

**Figure 1:**
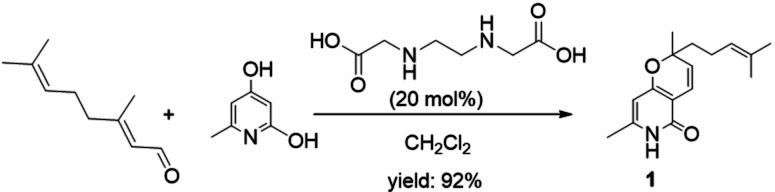
Synthesis of 2H‐pyran compound 1 starting from 6‐methylpyridine‐2,4‐diol and citral.

Here, 2H‐pyran compound 1 was synthesized from 6‐methylpyridine‐2,4‐diol and citral (Figure 1).

Applying the reaction conditions described in reference 27, our investigation started with the reactions of 2H‐pyran compound 1 and (4‐ethylphenyl) boronic acid 2a, and the desired product 3a could be afforded in 85 % yield (Table 1, entry 1). To improve the reaction yield, we further optimized the reaction conditions. Firstly, with Cu(OAc)_2_ as the catalyst, the desired product 3a was isolated with the yield of 27 % in the presence of K_2_CO_3_ (Table 1, entry 2). Then, other copper catalysts were used to promote this reaction. However, when the reactions were conducted in the presence of other Cu‐containing compounds such as CuO, CuSO_4_, CuBr_2_ and CuCl_2_, the highest yield of product 3a was merely up to 36 % (Table 1, entries 3–6). Hence, we turned to try Cu(I). A slightly higher yield (37 %) was achieved utilizing CuCl in DMF at room temperature (Table 1, entry 7). Higher yields were observed using Cu(I) catalysts such as CuBr, CuI, etc. (Table 1, entries 8−9). In particular, when the reaction was conducted with CuI, product 3a was obtained in a higher yield (45 %) (Table 1, entry 9). As a result, CuI was used as the catalyst in this study as described in ref [27]. Consequently, we examined the base for *N*‐arylation of this 2*H*‐pyran‐containing compounds. When the reaction was carried out in Et_3_N, a higher yield (57 %) was observed (Table 1, entry 10). However, when other bases such as DABCO, Pyridine, Na_2_CO_3_, Cs_2_CO_3_, K_3_PO_4_, KO^*t*^Bu were employed, all of them were proved to be inferior to Et_3_N in terms of yield (Table 1, entries 11–16). The effect of inorganic bases on promoting reactions were slightly worse than those of organic bases. To enhance the yield, the reaction was carried out with other solvents such as DCE, toluene, MeCN, DMAc, MeOH, EtOAc with CuI as the catalyst (Table 1, entries 17−22). However, all of them were proved to be inferior to DMSO in terms of yield (Table 1, entry 1) Finally, we tried to raise the temperature to increase the yield. However, increased temperature (40 °C) gave rise to an adverse effect on the yield of the product, reducing the product yield to 55 % (Table 1, entry 23). Higher temperature (>60 °C) led to the product 3a in a lower yield (Table 1, entries 24–25).

**Table 1:** Optimization of the reaction conditions.^[a]^

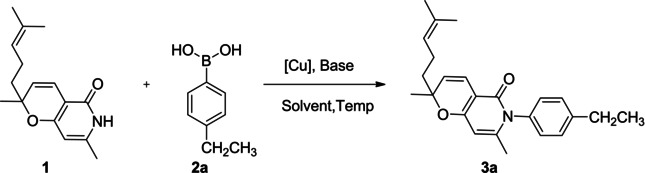					
Entry	[Cu]	Base	Solvent	Temp. (°C)	Yield (%)^[b]^
1	CuI	Et_3_N	DMSO	25	85
2	Cu(OAc)_2_	K_2_CO_3_	DMF	25	27
3	CuO	K_2_CO_3_	DMF	25	36
4	CuSO_4_	K_2_CO_3_	DMF	25	20
5	CuBr_2_	K_2_CO_3_	DMF	25	13
6	CuCl_2_	K_2_CO_3_	DMF	25	10
7	CuCl	K_2_CO_3_	DMF	25	37
8	CuBr	K_2_CO_3_	DMF	25	39
9	CuI	K_2_CO_3_	DMF	25	45
10	CuI	Et_3_N	DMF	25	57
11	CuI	DABCO	DMF	25	25
12	CuI	Pyridine	DMF	25	45
13	CuI	Na_2_CO_3_	DMF	25	32
14	CuI	Cs_2_CO_3_	DMF	25	36
15	CuI	K_3_PO_4_	DMF	25	35
16	CuI	KO^*t*^Bu	DMF	25	22
17	CuI	Et_3_N	DCE	25	15
18	CuI	Et_3_N	Toluene	25	36
19	CuI	Et_3_N	MeCN	25	20
20	CuI	Et_3_N	DMAc	25	18
21	CuI	Et_3_N	MeOH	25	trace
22	CuI	Et_3_N	EtOAc	25	26
23	CuI	Et_3_N	DMSO	40	55
24	CuI	Et_3_N	DMSO	60	40
25	CuI	Et_3_N	DMSO	80	30

^[a]^ Reaction conditions: **1 a** (0.5 mmol), **2 a** (0.6 mmol), [Cu] (0.2 equiv.), base (2.0 equiv.), solvent (5.0 mL), rt under air, 12 h. ^[b]^ Isolated yields.

To further investigate the scope of the Cu‐catalyzed *N*‐arylation of 2*H*‐pyran‐containing compounds, various arylboronic acids (2) were utilized as a coupling partner under the optimized conditions (**1** (0.5 mmol), **2** (0.6 mmol), CuI (0.2 equiv.), Et_3_N (2.0 equiv.), DMSO (5.0 mL), rt under air, 12 h) (Table 2). For a range of arylboronic acids, the corresponding coupling with compound **1** proceeded smoothly, giving the desired *N*‐arylation 2*H*‐pyran derivatives (**3**) with high efficiency and *regio*‐selectivity (*N*‐arylation not *O*‐arylation). When OMe‐substituted arylboronic acid was used, no desired coupling product 3 k was obtained, indicating that the reaction did not tolerate electron‐rich arylboronic acids. Thus, the efficiency of the reaction was sensitive to the substituent on the aromatic rings in different arylboronic acids. However, the same method was effective for a wide range of arylboronic acids with either electron‐rich or electron‐poor aryl groups when 2,4‐dihydroxyquinolines were used as the substrates.^[27]^ The reason for this experimental phenomenon may be due to the influence of the electronic effect of aromatic rings.

**Table 2:** Substrate screening.^[a]^

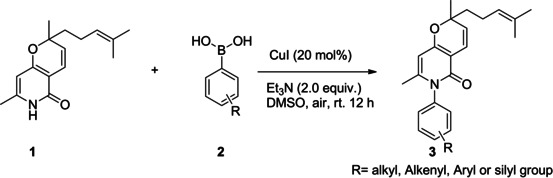
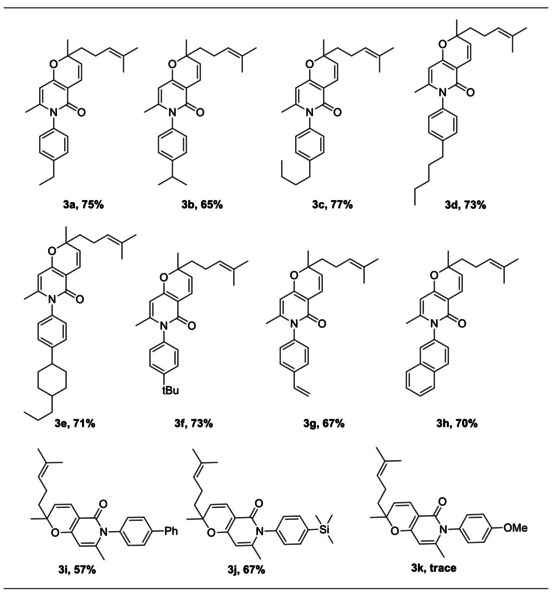

^[a]^ 1 (0.5 mmol), 2 (0.6 mmol), CuI (0.2 equiv.), Et_3_N (2.0 equiv.), DMSO (5.0 mL), rt under air, 12 h.

### Antitumor Activity of Citral Pyranopyridine Derivatives

The IC_50_ values of 10 citral pyrrolidone derivatives on HepG2 liver cancer cells were preliminarily measured using the WST‐8 method (Table 3) to evaluate their inhibitory activity on tumor cells. The IC_50_ values of these derivatives ranged from 5.3 μM to 38.0 μM. Using the IC_50_ value of cisplatin in the control group as the screening criterion, the IC_50_ values of these compounds were all below cisplatin. Among them, **3 h** showed the best inhibitory activity against HepG2 liver cancer cells, with an IC_50_ value of 5.3 μM.

**Table 3:** Determination of antitumor (HepG2 liver cancer cells) activity of citral pyranopyridine derivatives.

Compound	IC_50_/μM
**3 a**	7.4±0.07
**3 b**	16.5±0.03
**3 c**	34.2±0.08
**3 d**	25.1±0.05
**3 e**	23.0±0.72
**3 f**	13.1±0.50
**3 g**	38.0±0.03
**3 h**	5.3±1.20
**3 i**	9.6±0.05
**3 j**	12.6±0.06
cisplatin	6.5±0.40

### Inhibition of HepG2 Cell Apoptosis Mechanism by 3 h

**Figure 2:**
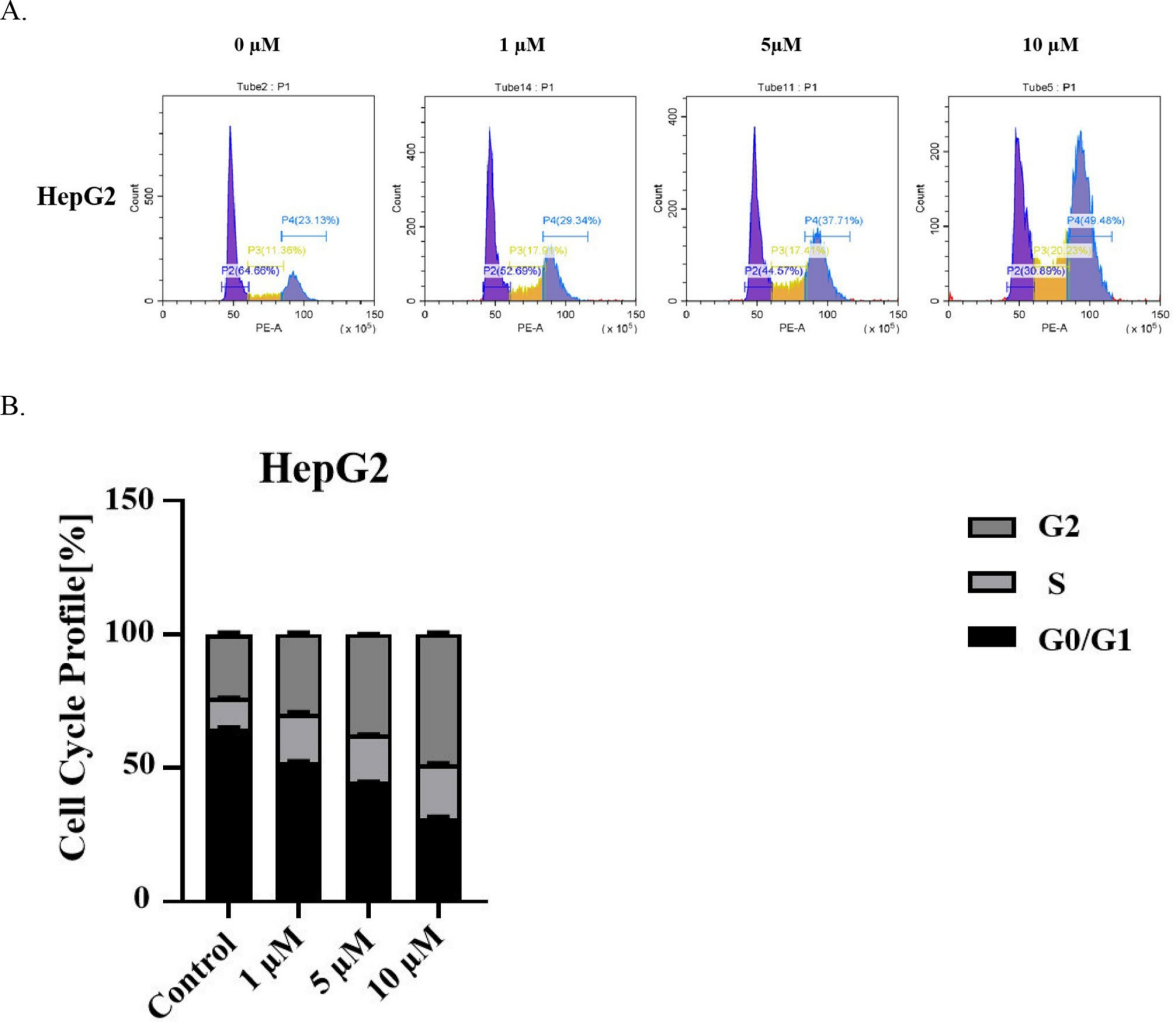
Effect of **3 h** on the HepG2 cell cycle in liver cancer cells (Note: A represents flow cytometry analysis of cell cycle; B represents statistical analysis of cell cycle).

Due to the best inhibitory effect of **3 h** on HepG2 liver cancer cells, we chose it as the research subject. The test results from NMR and HPLC indicated that the purity of **3 h** was 99.74 %. Different concentrations of **3 h** (1 μmol/L, 5 μmol/L, and 10 μmol/L) were used to treat HepG2 cells for 24 hours. Flow cytometry cell cycle analysis was shown in Figure 2A, and cell cycle statistical analysis was presented in Figure 2B. Compared with the control group, the proportion of G_0_/G_1_ phase cells gradually decreased with increasing concentrations of **3 h**, while the proportion of S phase and G2 phase cells gradually increased. This trend became more pronounced with higher concentrations of **3 h**, indicating that cell‐cycle arrest occurred in the S phase. 
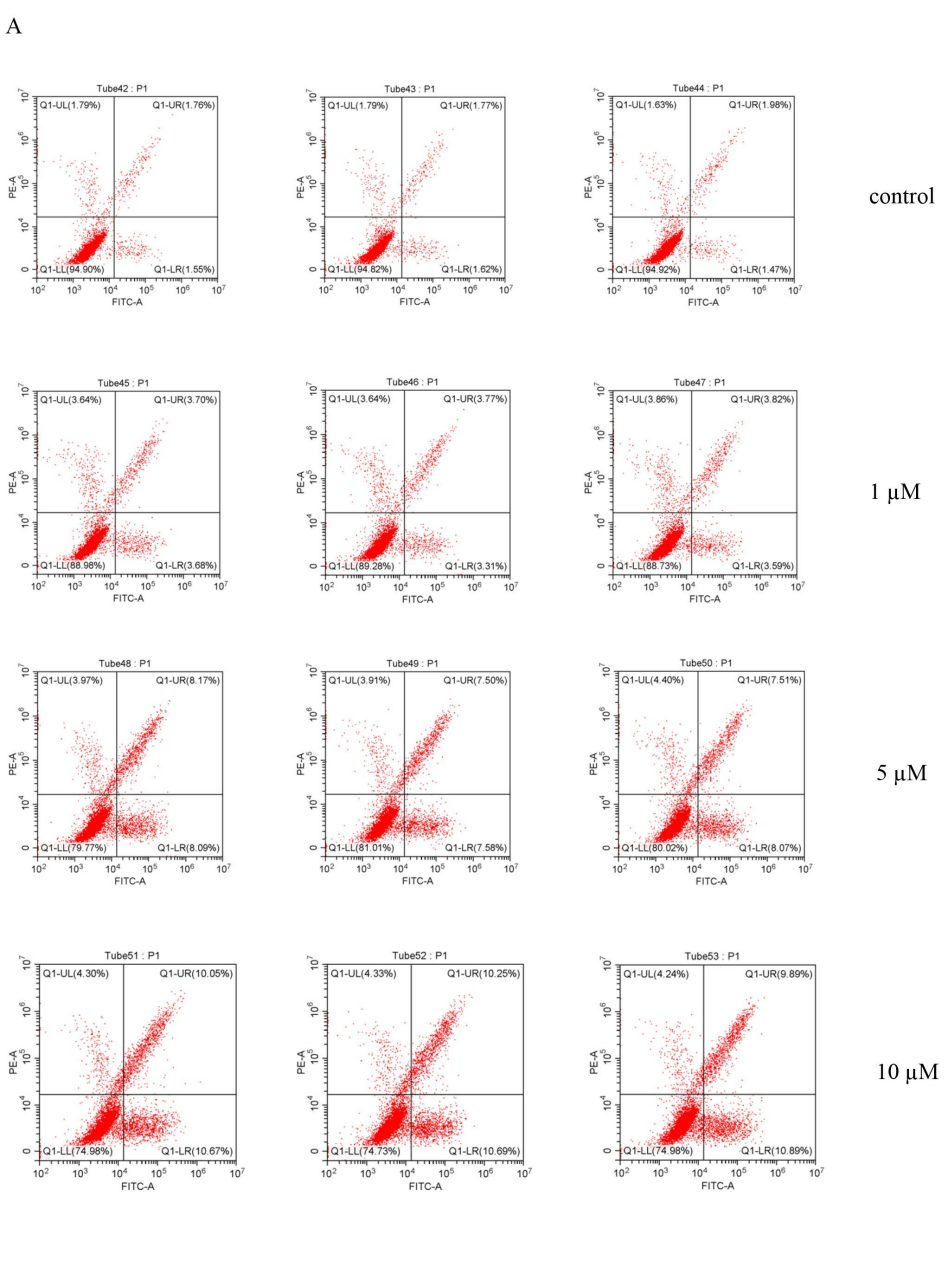


### Compound 3 h Promotes Apoptosis of HepG2 Cells

**Figure 3:**
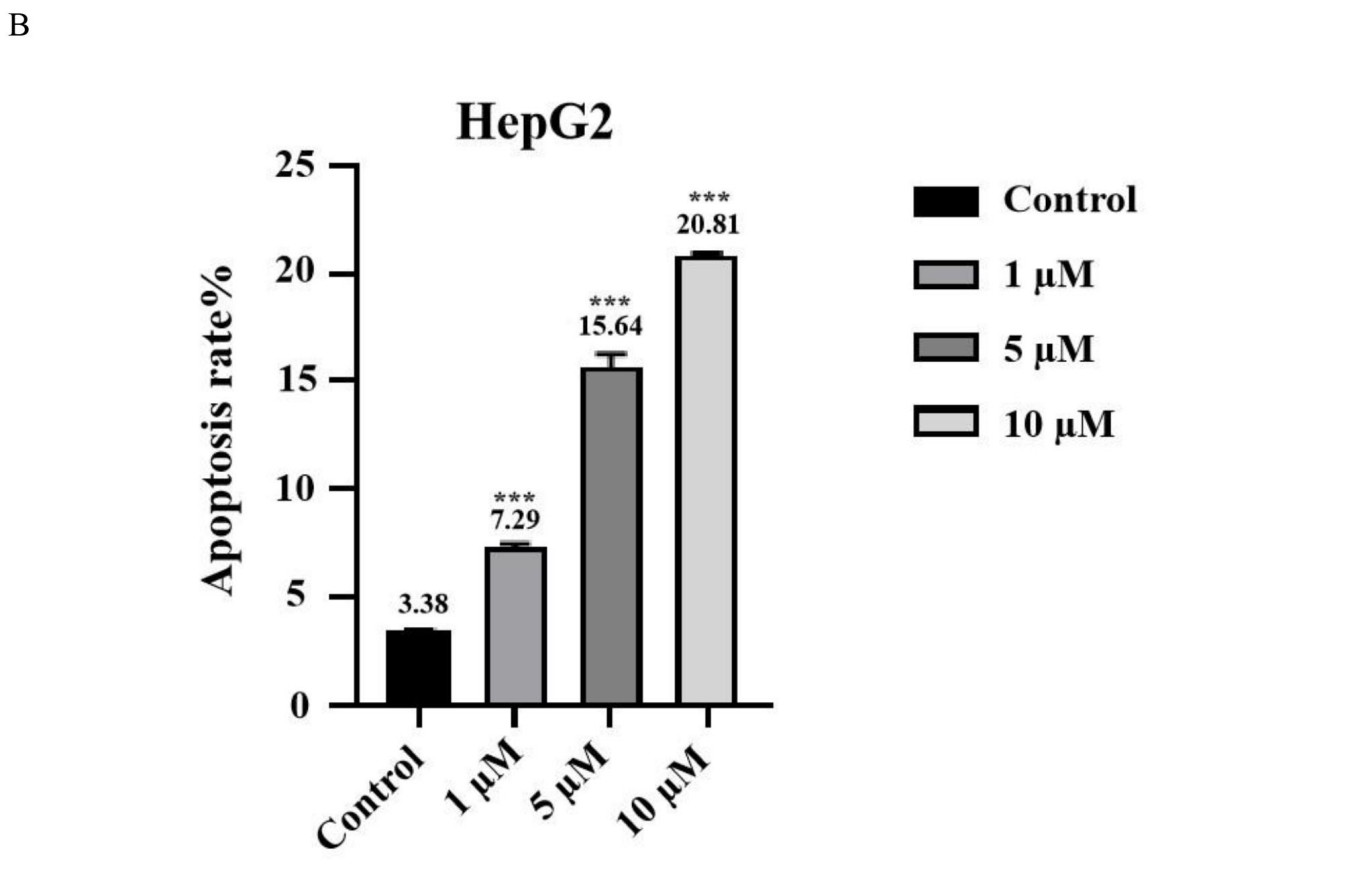
Apoptosis of HepG2 cells in liver cancer cells was determined by the Annexin V‐EGFP/PI method (A). Statistical analysis of HepG2 cells apoptosis in liver cancer cells (B) (Note: “*” represents *P*<0.05, “* *” represents *P*<0.01, and “* * *” represents *P*<0.001) (n=3).

Similarly, different concentrations of **3 h** were used to treat human liver cancer HepG2 cells for 24 hours, and the cell apoptosis rate was assessed using the Annexin V‐EGFP/PI method. A total of three tests were conducted (Figure 3A). The early apoptosis rates induced by different concentrations of **3 h** (1 μmol/L, 5 μmol/L, and 10 μmol/L) were 7.29 %, 15.64 %, and 20.81 % of HepG2 cells, respectively. And all of which were higher than the control group without drug treatment (3.38 %) (***P<0.001) (Figure 3B). Compound **3 h** induces apoptosis in HepG2 cells in a dosage‐dependent manner.

**Figure 4:**
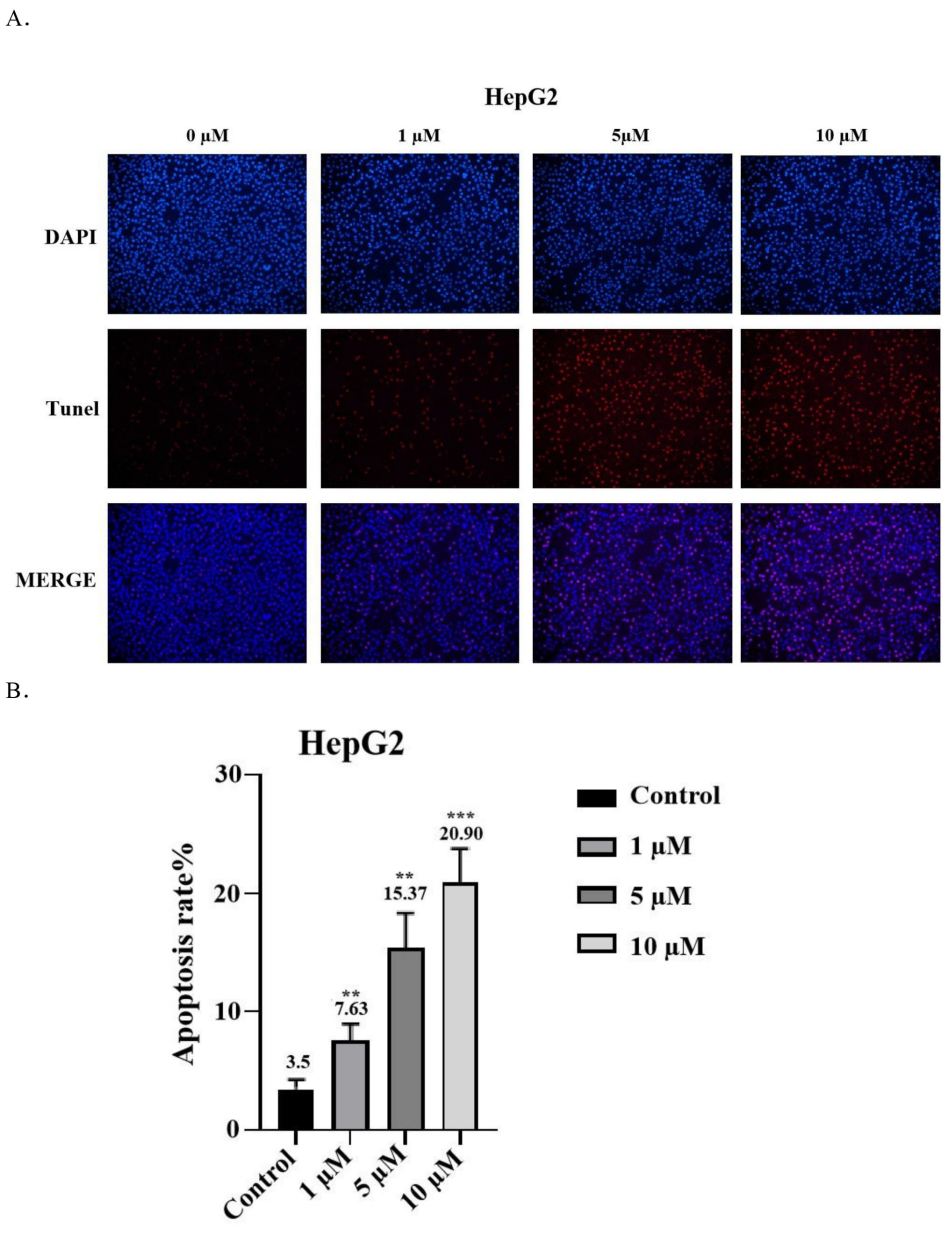
Apoptosis of HepG2 cells was determined using a TUNEL assay (Note: A represents the TUNEL method for detecting HepG2 cell apoptosis, and B represents statistical analysis of cell apoptosis) (n=3).

To further determine the apoptotic effect of **3 h** on human liver cancer HepG2 cells after 24 hours of treatment, the TUNEL method was used to detect cell apoptosis. The results showed that the apoptosis rate in the control group was 3.50 % (Figure 4). At a treatment concentration of 1 μmol/L, the apoptosis rate reached 7.63 %. When the treatment concentrations were increased to 5 μmol/L and 10 μmol/L, the apoptosis rates were 15.37 % and 20.90 %, respectively. These results were consistent with the overall findings from the Annexin V‐EGFP/PI method, indicating that **3 h** has a significant pro‐apoptotic effect on HepG2 cells.

### Mechanism Underlying the Inhibition of HepG2 Cell Proliferation and Promotion of Cell Apoptosis by 3 h

**Figure 5:**
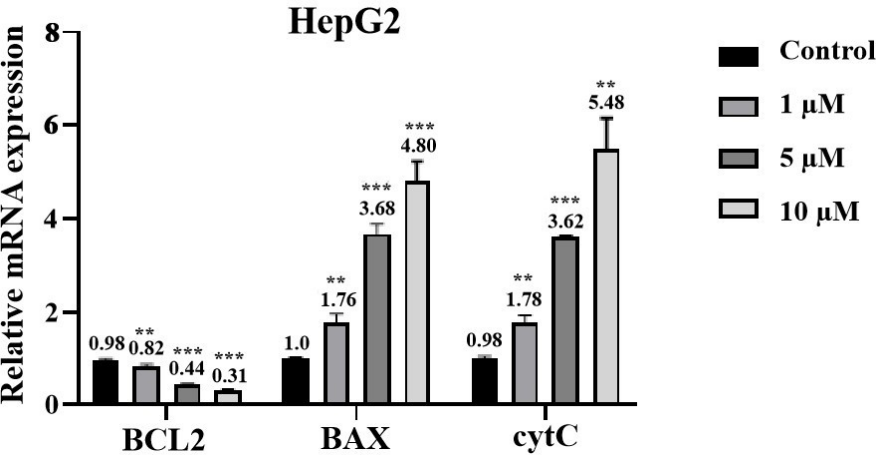
The expression of apoptosis‐related genes was determined by real‐time PCR.

Furthermore, real‐time fluorescence quantitative PCR was used to detect the expression levels of *BAX*, *BCL2* and cytochrome c (*cytC*) (Figure 5). The relative protein expression levels of BAX were 1.76, 3.68, and 4.80 when the cells were stimulated with different concentrations of **3 h** (1 μmol/L, 5 μmol/L, and 10 μmol/L), respectively. The relative expression levels of *cytC* were 1.78, 3.62 and 5.48, respectively, all of which were higher than those in the control group. Correspondingly, the relative expression levels of *BCL2* were 0.82, 0.44, and 0.31, respectively, all of which were lower than those in the control group, showing a down‐regulation trend with the increased concentration of **3 h**.

**Figure 6:**
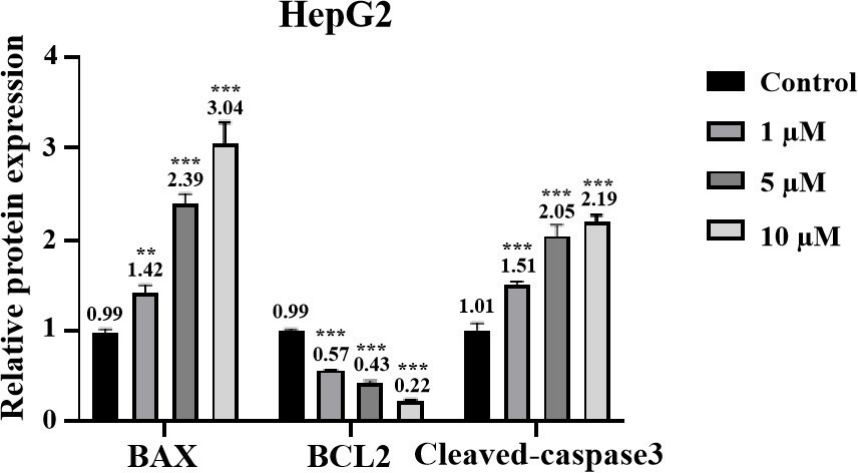
Expression levels of apoptosis‐related proteins in HepG2 cells (Note: A. Immunoblotting to detect the expression of apoptosis related proteins; B. Statistical analysis of expression levels of apoptosis related proteins) (n=3).

Besides, Western blot analysis was used to detect the protein levels of BAX, BCL2 and Cleaved‐Caspase3 (Figures 6A, B). The results showed that, following treatment with 1 μmol/L, 5 μmol/L and 10 μmol/L of **3 h**, the protein expression levels of BAX in HepG2 cells were 1.42, 2.39 and 3.04, respectively. The protein expression levels of Cleaved caspase‐3 were 1.51, 2.05 and 2.19, suggesting that **3 h** also increased the protein expression levels of BAX and Cleaved‐Caspase3. In contrast, the protein expression levels of BCL2 were 0.57, 0.43 and 0.22, indicating a down‐regulation trend. BAX and BCL‐2, affect apoptosis primarily by binding to mitochondrial membranes,^[28]^ which means that **3 h** may be involved in apoptosis of multiple apoptotic pathways. However, how **3 h** affects apoptosis requires further research.

**Figure 7:**
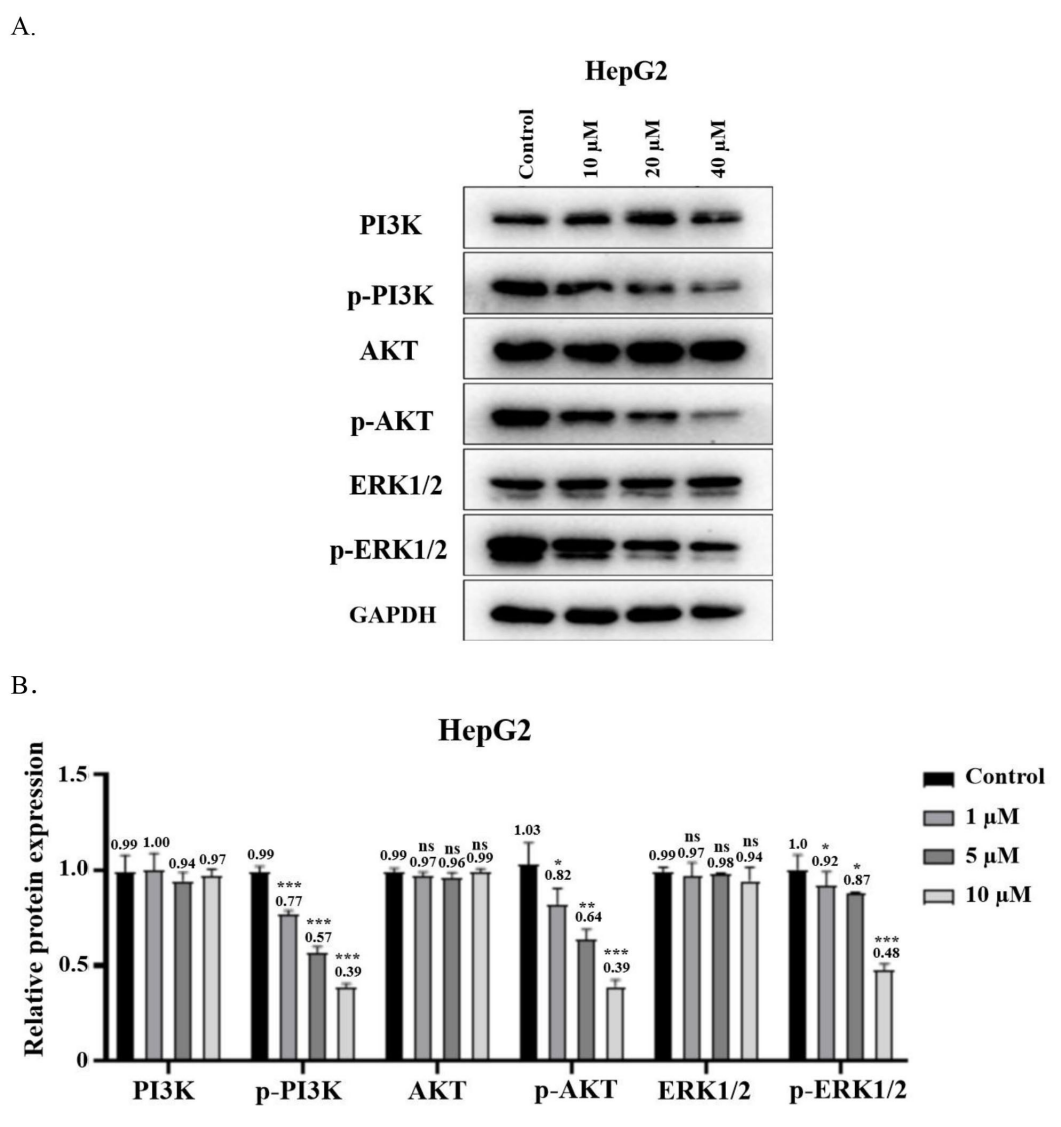
Expression levels of key proteins of cell signaling pathways were determined by immunoblotting.

To further explore the molecular mechanism of **3 h**, the changes in key proteins of the MAPK and PI3 K/AKT signaling pathways were analyzed using Western blotting (Figure 7). Compared with the control group, the expression levels of PI3 K, ATK, and ERK1/2 in HepG2 cells after treatment with **3 h** showed no significant changes, while the expression levels of p‐PI3 K, p‐AKT, and p‐ERK were inhibited. This indicated that **3 h** may suppress cell proliferation by down‐regulation the protein expression levels in the MAPK and PI3 K/AKT pathways. The PI3 K/AKT pathway is closely related to apoptosis, and many previous experiments have confirmed that in HePG2 cells, PI3 K/AKT is primarily involved in apoptosis caused by cellular stress. It is an important pathway in cancer development.^[29]^ Our experiments have confirmed that **3 h** significantly affects the phosphorylation levels of PI3 K and AKT, indicating that **3 h** influences the apoptosis pathway by modulating PI3 K and AKT. This was significant for the potential use of **3 h** as a new model for drug therapy. ERK can be regulated by MAPK to induce apoptosis,^[30]^ however, we have not measured the level of other MAPK‐related proteins. Since MAPK is a large family of proteins, the process of apoptosis regulated by MAPK through ERK is complex and requires further investigation.

## Conclusions

In summary, citral derivatives were designed and synthesized to investigate their inhibitory activities in HepG2 cells. It was found that the IC_50_ value of **3 h** was the lowest, indicating the best inhibitory activity against tumor cells. Cell cycle experiments confirmed that cell‐cycle arrest occurred in the S phase. Furthermore, Annexin V/PI double staining and TUNEL assay experiments showed that **3 h** (one of the newly synthesized citral derivatives) could significantly promote apoptosis in a dosage‐dependent manner. Fluorescence real‐time quantitative PCR and Western blotting experiments were used to further demonstrate that **3 h** was involved in the MAPK and PI3 K/AKT signaling pathways. Briefly, **3 h** possessed inhibitory effects on HepG2 liver cancer cells and may have great potential for anti‐cancer drug screening in the future.

## Materials and Methods

### General Information

All reagents were purchased from Shanghai Aladdin Bio‐Chem Technology Co., Ltd. (Shanghai, China), unless noted otherwise, and were used without purification. Purification of products was conducted by flash chromatography on silica gel (200–300 mesh). Nuclear magnetic resonance (NMR) spectra were measured on a Bruker Avance III 400 (Bruker, Billerica, MA, USA). The ^1^ *H*‐NMR (400 MHz) chemical shifts were obtained relative to CDCl_3_ as the internal reference (CDCl_3_: δ 7.26 ppm). The ^13^C‐NMR (100 MHz) chemical shifts were obtained using CDCl_3_ as the internal standard (CDCl_3_: δ 77.16 ppm). Chemical shifts were reported in ppm using tetramethylsilane as the internal standard (s=singlet, d=doublet, t=triplet, q=quartet, dd=doublet of doublets, m=multiplet). HR‐MS data were obtained using a VG ZAB‐HS mass spectrometer and a Bruker Apex IV FTMS spectrometer.

### General Procedure for the N‐Arylation of Pyranopyridines with Boronic Acids

To a mixture of pyranopyridine (0.5 mmol), arylboronic acid 2 (0.6 mmol), CuI (20 mmol %), Et_3_N (2.0 equiv.) was added dimethyl sulfoxide (5.0 mL) at room temperatureunder under atmosphere. The reaction was stirred in an IKA magneticstirring heating module at room temperature for 12 h. After completion, water and ethyl acetate were added to the mixture. The organic layer was separated, and the aqueous layer was extracted with ethyl acetate. The organic layers were combined. Then, organic layers were washed with brine and dried over Na_2_SO_4_, filtered, and concentrated in vacuo. The residue was purified by flash chromatography eluting with petroleum ether and ethyl acetate (PE/EA=5 : 1) to afford the corresponding products.

### Cell Culture Method

HepG2 liver cancer cells, purchased from ATCC, Ltd., were cultured in DMEM medium (containing 10 % inactivated fetal bovine serum and 1 % penicillin/streptomycin) under 5 % CO_2_ conditions in a 37 °C constant temperature cell culture incubator.

After cell rejuvenation, the cell suspension was transferred to a 6 cm culture dish and placed in a 37 °C, 5 % CO_2_ constant temperature incubator for further culture.

During serial passagings of HepG2 liver cancer cells, 0.25 % trypsin was used to digest the adherent cells. After some cells detached, 3 mL of DMEM medium containing serum was added to terminate digestion. The upper liquid was collected after centrifugation and inoculated into a new culture dish for further culture. The cell line was tested for mycoplasma contamination using the EEZ‐PCR mycoplasma detection kit (20‐700‐20, BI company).

Finally, the cell count was performed. The counting board and cover glasses were washed and wiped clean with 75 % ethanol disinfectant, dried, and an appropriate amount of cell suspension was aspirated, diluted by a certain multiple, and added to the blood cell counting board. The board was placed under an inverted microscope to observe the cell distribution. The concentration of the cell suspension was calculated using the formula: concentration of cell suspension=(number of cells in the four large squares of the counting board/4)×dilution multiple×10^4^ ells/mL.

### Drug Preparation

Preparation: to prepare a 10 mM of drug DMSO solution, and to be filtered using a 0.22 μm filter membrane, then stored at −20 °C for further experiment.

### Annexin V‐EGFP/PI Cell Apoptosis Detection

Cancer cells in the logarithmic growth phase were inoculated into a six‐well plate and incubated at 37 °C under 5 % CO_2_ conditions. Twenty‐four hours later, the cells were digested with trypsin and collected by centrifugation for 5 minutes, then re‐suspended in 100 μL of pre‐cooled Annexin V binding buffer, and 5 μL of Annexin V‐EGFP and 5 μL of PI. The cells were mixed and incubated at room temperature in the dark. After 15 minutes, 400 μL of pre‐cooled Annexin V binding buffer was added, and the cells were gently mixed again. Finally, the cells were collected and detected using the flow cytometer. The data were recorded and analyzed using Modi Fit software.

### Cell Apoptosis Detection Using TUNEL Method

For TUNEL assays, the cells were washed once with PBS and fixed with 4 % paraformaldehyde for 60 minutes. 50 μL of TUNEL detection solution were added to the sample, which was then incubated at 37 °C in the dark for 60 minutes. During incubation, the surrounding paper or cotton wool was kept moist with water to minimize the evaporation of the TUNEL detection solution. After washing three times with PBS, the sample was observed under a fluorescence microscope.

### Cell Cycle Detection

Cancer cells were treated with different concentrations of citral derivatives for 24 hours. The cells were washed twice with PBS buffer. Then, 1 mL of pre‐cooled 70 % ethanol was added, and the cells were gently mixed. The cells were incubated at 4 °C for 2 hours. The cells were centrifuged for 3 minutes, and the sample supplemented with 2 μL of propidium iodide staining solution and 2 μL of RNase A. The sample was slowly resuspended and fully precipitated. Finally, the cells were incubated at 37 °C in the dark for 30 minutes. The cell cycle was detected using a flow cytometer.

### Real‐time Fluorescence Quantitative PCR Analysis

Total RNA was extracted using RNA extraction kit according to the operation manual, and then mRNA was reversed to cDNA using reverse transcription kit. Real‐time fluorescence quantitative PCR was used to detect the relative expression levels of apoptosis‐related genes *BAX*, *BCL2*, *cytochrome C*, and *Caspase3*. The expression level was calculated according to the formula 2^−ΔΔCt^. The primer sequences used in this study were shown in Supporting Information.

### Western Blot

Following treatment with different concentrations of drugs, the cancer cells were collected by centrifugation, and then the supernatant was discarded. The cells were washed three times with PBS, the supernatant was discarded after centrifugation. 500 μL of RIPA cell lysis buffer was added for lysis on ice for 30 minutes. Once the cells were fully lysed, the mixture was centrifuged, and the protein sample was collected from the supernatant. The protein concentration was measured at 562 nm using the BCA method. After SDS‐PAGE electrophoresis, the protein bands were transferred to a PVDF membrane. Following labeling, the membrane was blocked with 5 % skim milk powder for 1 hour. Primary antibodies were then added to detect Bax, Bcl‐2, cleaved caspase‐3, PI3 K, p‐PI3 K, AKT, p‐AKT, ERK1/2, p‐ERK1/2 and β‐actin, and the membrane was incubated overnight at 4 °C. After the overnight incubation, the membrane was washed three times with PBST, followed by the addition HRP‐labeled secondary antibodies and incubation at room temperature for 1 hour. The membrane was washed three times with PBST, and imaged using an immunoblot imaging system. The results were analyzed by Image J to obtain the gray value.

### Antiproliferative Assays

HepG2 cells were inoculated into 96‐well cell culture plates. After drug treatment, 10 μL MTT solution (5 mg/mL) was added to each well, and the culture was continued for 4 hours. The culture medium was then carefully removed, taking care not to disturb the purple crystals at the bottom. 100 μL DMSO was added to each well and shaken for 10 minutes to fully dissolve the purple crystal. The absorbance value (OD value) of each well was measured by enzyme‐labeled instrument at the wavelength of 490 nm or 570 nm.

### Statistical Analysis

Statistical software SPSS20.0 was used for analysis. The data of each experimental group were expressed as mean±standard deviation (means±S.E.M). Differences between groups were presented using * to indicate statistical significance, with P<0.05 indicating statistical difference. *represents P<0.05, **represents P<0.01 and ***represents P<0.001.

## Acknowledgement

This research was funded by the Key R & D Program of Jiangxi Science and Technology Department (20212bbf63046), the Training Program for Academic and Technical Leaders of Major Discipline in Jiangxi Province‐Young Talent Project (20212bcj23013), the development and commercialization of the camphor tree essential oil source (i) (2020‐05‐02), the synthesis of heterocyclic derivatives and their inhibitory activities against anthracnose of camellia (innovation project 2022: 26), and the research on the antibacterial activity of essential oil‐based derivatives in the PhD startup project of the Jiangxi Academy of Forestry (2022522701).

## Conflict of Interests

The authors declare no conflict of interest.

1

## Supporting information

As a service to our authors and readers, this journal provides supporting information supplied by the authors. Such materials are peer reviewed and may be re‐organized for online delivery, but are not copy‐edited or typeset. Technical support issues arising from supporting information (other than missing files) should be addressed to the authors.

Supporting Information

## Data Availability

The data that support the findings of this study are available in the supplementary materials of this article.
