# Supplementary material for: Newly Synthesized Citral Derivatives Serve as Novel Inhibitor in HepG2 Cells
Source: ChemistryOpen. 2024 Nov 26;14(4):e202400112. doi: 10.1002/open.202400112 (PMC11973499; doi:10.1002/open.202400112)
Supplement: Supplementary file 1 — Supporting Information [file OPEN-14-e202400112-s001.pdf]

ChemistryOpen

Supporting Information

Newly Synthesized Citral Derivatives Serve as Novel Inhibitor in HepG2 Cells

Wei Gao, Xiaoju Hua, Shengliang Liao, Zhikai Xiahou, Haikuan Yang, Lifang Hu,* and Yunyang Chi*

Newly Designed Citral Derivatives Serve as Novel HepG2 Cells inhibitor

Wei Gao, Xiaoju Hua, Shengliang Liao, Zhikai Xiahou, Haikuan Yang, Lifang Hu* and Yunyang Chi *

Supporting Information

Characterization Data for Products 3a–3j

6-(4-ethylphenyl)-2,7-dimethyl-2-(4-methylpent-3-en-1-yl)-2H-pyrano[3,2-c]pyridin-5(6H)-one (3a). Yellow solid, 75% yield (eluent: PE/EA = 5:1). ^{1}H NMR (400 MHz, $CDCl_3$) δ 7.32-7.27 (m, 2H), 7.12-7.04 (m, 2H), 6.63 (d, J = 10.0 Hz, 1H), 5.80 (s, 1H), 5.31 (d, J = 10.0 Hz, 1H), 5.15-5.08 (m, 1H), 2.70 (q, J = 7.6 Hz, 2H), 2.11 (q, J = 7.6 Hz, 2H), 1.91 (s, 3H), 1.82-1.74 (m, 1H), 1.68 (s, 3H), 1.66-1.61 (m, 1H), 1.60 (s, 3H), 1.42 (s, 3H), 1.27 (t, J = 7.6 Hz, 3H). ^{13}C NMR (100 MHz, $CDCl_3$) δ 162.1, 161.2, 146.1, 144.6, 136.3, 131.8, 129.0, 128.0, 123.9, 123.3, 118.3, 104.3, 100.2, 80.9, 41.8, 28.5, 27.3, 25.7, 22.7, 21.7, 17.7, 15.2. HRMS (ESI): *m/z* $[M+H]^+$calcd for $C_{23}H_{28}NO_2^+$: 350.2115, found: 350.2111.

6-(4-isopropylphenyl)-2,7-dimethyl-2-(4-methylpent-3-en-1-yl)-2H-pyrano[3,2-c]pyridin-5(6H)-one (3b). Yellow solid, 65% yield (eluent: PE/EA = 5:1).^{1}H NMR (400 MHz, $CDCl_3$) δ 7.35-7.29 (m, 2H), 7.11-7.04 (m, 2H), 6.62 (d, J = 10.0 Hz, 1H), 5.80 (s, 1H), 5.31 (d, J = 10.0 Hz, 1H), 5.11 (t, J = 7.2 Hz, 1H), 2.95 (dq, J = 14.0, 6.8 Hz, 1H), 2.11 (q, J = 7.6 Hz, 2H), 1.91 (s, 3H), 1.83-1.74 (m, 1H), 1.68 (s, 3H), 1.67-1.63 (m, 1H), 1.60 (s, 3H), 1.42 (s, 3H), 1.29 (s, 3H), 1.27 (s, 3H). ^{13}C NMR (100 MHz, $CDCl_3$) δ 162.0, 161.2, 149.2, 146.1, 136.4, 131.8, 127.9, 127.6, 123.9, 123.3, 118.3, 104.3, 100.2, 80.9, 41.8, 33.8, 27.3, 25.6, 23.9, 22.7, 21.7, 17.6. HRMS (ESI): *m/z* $[M+H]^+$calcd for $C_{24}H_{30}NO_2^+$: 364.2271, found: 364.2276.

6-(4-butylphenyl)-2,7-dimethyl-2-(4-methylpent-3-en-1-yl)-2H-pyrano[3,2-c]pyridin-5(6H)-one (3c). Yellow solid, 77% yield (eluent: PE/EA = 5:1). ^{1}H NMR (400 MHz, $CDCl_3$) δ 7.30-7.25 (m, 2H), 7.09-7.03 (m, 2H), 6.63 (d, J = 10.0 Hz, 1H),

5.80 (s, 1H), 5.31 (d, J = 10.0 Hz, 1H), 5.11 (m, 1H), 2.69-2.61 (m, 2H), 2.11 (q, J = 7.6 Hz, 2H), 1.90 (s, 3H), 1.80-1.74 (m, 2H), 1.68 (s, 3H), 1.65-1.63 (m, 2H), 1.60 (s, 3H), 1.42 (s, 3H), 1.40-1.33 (m, 2H), 0.94 (t, J = 7.2 Hz, 3H). ^{13}C NMR (100 MHz, $CDCl_3$) δ 162.0, 161.2, 146.1, 143.3, 136.3, 131.8, 129.5, 127.9, 123.9, 123.3, 118.3, 104.3, 100.2, 80.9, 41.8, 35.3, 33.3, 27.3, 25.7, 22.7, 22.4, 21.7, 17.6, 13.9. HRMS (ESI): *m/z* $[M+H]^+$calcd for $C_{25}H_{32}NO_2^+$: 378.2428, found: 378.2423.

2,7-dimethyl-2-(4-methylpent-3-en-1-yl)-6-(4-pentylphenyl)-2H-pyrano[3,2-c]pyridin-5(6H)-one (3d). Yellow solid, 73% yield (eluent: PE/EA = 5:1). ^{1}H NMR (400 MHz, $CDCl_3$) δ 7.29-7.25 (m, 2H), 7.09-7.03 (m, 2H), 6.63 (d, J = 10.0 Hz, 1H), 5.80 (s, 1H), 5.31 (d, J = 10.0 Hz, 1H), 5.11 (t, J = 7.2 Hz, 1H), 2.67-2.61 (m, 2H), 2.11 (q, J = 7.6 Hz, 2H), 1.91 (s, 3H), 1.89-1.73 (m, 1H), 1.68 (s, 3H), 1.65-1.62 (m, 3H), 1.60 (s, 3H), 1.42 (s, 3H), 1.40-1.30 (m, 4H), 0.90 (t, J = 6.8 Hz, 3H). ^{13}C NMR (100 MHz, $CDCl_3$) δ 162.1, 161.2, 146.1, 143.4, 136.3, 131.9, 129.5, 127.9, 123.9, 123.4, 118.3, 104.3, 100.2, 80.9, 41.8, 35.6, 31.5, 30.9, 27.3, 25.6, 22.7, 22.5, 21.7, 17.6, 14.0. HRMS (ESI): *m/z* $[M+H]^+$calcd for $C_{26}H_{34}NO_2^+$: 392.2584, found: 392.2580.

2-methyl-2-(4-methylpent-3-en-1-yl)-6-(4-(4-propylcyclohexyl)phenyl)-2H-pyrano[3,2-c]pyridin-5(6H)-one (3e). Yellow solid, 71% yield (eluent: PE/EA = 5:1). ^{1}H NMR (400 MHz, $CDCl_3$) δ 7.33-7.27 (m, 2H), 7.11-7.03 (m, 2H), 6.62 (d, J = 10.0 Hz, 1H), 5.79 (s, 1H), 5.30 (d, J = 10.0 Hz, 1H), 5.11 (t, J = 7.2 Hz, 1H), 2.56-2.46 (m, 1H), 2.11 (q, J = 7.6 Hz, 2H), 1.96-1.93 (m, 1H), 1.90 (s, 3H), 1.87-1.85 (m, 1H), 1.82-1.73 (m, 2H), 1.68 (s, 3H), 1.65 (m, 1H), 1.60 (s, 3H), 1.51-1.43 (m, 2H), 1.42 (s, 3H), 1.37-1.32 (m, 2H), 1.31-1.28 (m, 2H), 1.24-1.19 (m, 2H), 1.11-1.00 (m, 2H), 0.90 (t, J

= 7.2 Hz, 3H). ^{13}C NMR (100 MHz, $CDCl_3$) δ 162.0, 161.2, 148.2, 146.2, 136.4, 131.8, 127.9, 127.9, 123.9, 123.3, 118.3, 104.3, 100.2, 80.9, 44.3, 41.8, 39.7, 37.0, 34.3, 33.5, 27.3, 25.7, 22.7, 21.7, 20.0, 17.6, 14.4. HRMS (ESI): *m/z* $[M+H]^+$calcd for $C_{34}H_{40}NO_2^+$: 446.3054, found: 446.3051.

6-(4-(tert-butyl)phenyl)-2,7-dimethyl-2-(4-methylpent-3-en-1-yl)-2H-pyrano[3,2-c]pyridin-5(6H)-one (3f). Yellow solid, 73% yield (eluent: PE/EA = 5:1). ^{1}H NMR (400 MHz, $CDCl_3$) δ 7.51-7.43 (m, 2H), 7.11-7.05 (m, 2H), 6.63 (d, J = 10.0 Hz, 1H), 5.80 (s, 1H), 5.31 (d, J = 10.0 Hz, 1H), 5.12 (ddd, J = 7.2, 6.0, 1.2 Hz, 1H), 2.11 (q, J = 7.6 Hz, 2H), 1.91 (s, 3H), 1.82-1.74 (m, 1H), 1.68 (s, 3H), 1.67-1.62 (m, 1H), 1.60 (s, 3H), 1.42 (s, 3H), 1.34 (s, 9H). ^{13}C NMR (100 MHz, $CDCl_3$) δ 162.0, 161.1, 151.4, 146.2, 136.1, 131.8, 127.6, 126.5, 123.9, 123.3, 118.3, 104.2, 100.2, 80.9, 41.8, 34.7, 31.4, 27.3, 25.7, 22.7, 21.7, 17.7. HRMS (ESI): *m/z* $[M+H]^+$calcd for $C_{25}H_{32}NO_2^+$: 378.2428, found: 378.2424.

2,7-dimethyl-2-(4-methylpent-3-en-1-yl)-6-(4-vinylphenyl)-2H-pyrano[3,2-c]pyridin-5(6H)-one (3g). Yellow solid, 67% yield (eluent: PE/EA = 5:1). ^{1}H NMR (400 MHz, $CDCl_3$) δ 7.54-7.47 (m, 2H), 7.16-7.11 (m, 2H), 6.80-6.69 (m, 1H), 6.62 (d, J = 10.0 Hz, 1H), 5.84-5.75 (m, 2H), 5.32 (d, J = 10.0 Hz, 2H), 5.11 (t, J = 7.2 Hz, 1H), 2.11 (q, J = 7.6 Hz, 2H), 1.92 (s, 3H), 1.82-1.74 (m, 1H), 1.68 (s, 3H), 1.66-1.62 (m, 1H), 1.60 (s, 3H), 1.42 (s, 3H). ^{13}C NMR (100 MHz, $CDCl_3$) δ 162.0, 161.3, 145.8, 138.1, 138.0, 135.9, 131.9, 128.4, 127.3, 123.9, 123.5, 118.2, 115.2, 104.3, 100.5, 81.0, 41.8, 27.3, 25.7, 22.7, 21.6, 17.7. HRMS (ESI): *m/z* $[M+H]^+$calcd for $C_{23}H_{26}NO_2^+$: 348.1958, found: 348.1955.

2,7-dimethyl-2-(4-methylpent-3-en-1-yl)-6-(naphthalen-2-yl)-2H-pyrano[3,2-c]pyridin-5(6H)-one (3h). Yellow solid, 70% yield (eluent: PE/EA = 5:1). ^{1}H NMR (400 MHz, $CDCl_3$) δ 7.95 (d, *J* = 8.8 Hz, 1H), 7.92-7.87 (m, 1H), 7.86-7.80 (m, 1H), 7.68 (s, 1H), 7.56-7.48 (m, 2H), 7.27 (d, *J* = 8.4 Hz, 1H), 6.65 (d, *J* = 10.0 Hz, 1H), 5.85 (s, 1H), 5.33 (d, *J* = 10.0 Hz, 1H), 5.13 (t, *J* = 7.2 Hz, 1H), 2.13 (q, *J* = 7.6 Hz, 2H), 1.93 (s, 3H), 1.85-1.76 (m, 1H), 1.69 (s, 3H), 1.68-1.64 (m, 1H), 1.62 (s, 3H), 1.44 (s, 3H). ^{13}C NMR (100 MHz, $CDCl_3$) δ 162.1, 161.3, 146.0, 136.3, 133.6, 133.0, 131.9, 129.6, 128.1, 127.8, 127.1, 126.8, 126.6, 125.9, 123.9, 123.5, 118.2, 104.4, 100.5, 81.1, 41.8, 27.3, 25.7, 22.7, 21.7, 17.7. HRMS (ESI): *m/z* $[M+H]^+$calcd for $C_{25}H_{26}NO_2^+$: 372.1958, found: 372.1954.

6-([1,1'-biphenyl]-4-yl)-2,7-dimethyl-2-(4-methylpent-3-en-1-yl)-2H-pyrano[3,2-c]pyridin-5(6H)-one (3i). Yellow solid, 57% yield (eluent: PE/EA = 5:1). ^{1}H NMR (400 MHz, $CDCl_3$) δ 7.72-7.66 (m, 2H), 7.64-7.58 (m, 2H), 7.48-7.42 (m, 2H), 7.37 (dd, *J* = 8.4, 6.2 Hz, 1H), 7.27-7.23 (m, 2H), 6.64 (d, *J* = 10.0 Hz, 1H), 5.84 (s, 1H), 5.33 (d, *J* = 10.0 Hz, 1H), 5.12 (t, *J* = 7.2 Hz, 1H), 2.12 (q, *J* = 7.6 Hz, 2H), 1.97 (s, 3H), 1.84-1.75 (m, 1H), 1.69 (s, 3H), 1.66-1.63 (m, 1H), 1.61 (s, 3H), 1.43 (s, 3H). ^{13}C NMR (100 MHz, $CDCl_3$) δ 162.0, 161.3, 145.9, 141.6, 140.3, 138.0, 131.9, 128.8, 128.6, 128.3, 127.7, 127.3, 123.9, 123.5, 118.2, 104.3, 100.4, 81.0, 41.8, 27.3, 25.7, 22.7, 21.7, 17.7. HRMS (ESI): *m/z* $[M+H]^+$calcd for $C_{27}H_{28}NO_2^+$: 398.2115, found: 398.2111.

2,7-dimethyl-2-(4-methylpent-3-en-1-yl)-6-(4-(trimethylsilyl)phenyl)-2H-pyrano[3,2-c]pyridin-5(6H)-one (3j). Yellow solid, 67% yield (eluent: PE/EA = 5:1).

^1H NMR (400 MHz, $CDCl_3$) δ 7.66-7.57 (m, 2H), 7.18-7.11 (m, 2H), 6.62 (d, J = 10.0 Hz, 1H), 5.81 (s, 1H), 5.31 (d, J = 10.0 Hz, 1H), 5.11 (t, J = 7.2 Hz, 1H), 2.11 (q, J = 7.6 Hz, 2H), 1.91 (s, 3H), 1.82-1.75 (m, 1H), 1.68 (s, 3H), 1.67-1.64 (m, 1H), 1.60 (s, 3H), 1.42 (s, 3H), 0.29 (s, 9H). ^{13}C NMR (100 MHz, $CDCl_3$) δ 163.1, 162.3, 147.0, 142.4, 140.4, 135.7, 133.0, 128.5, 125.1, 124.5, 119.4, 105.4, 101.4, 82.1, 42.9, 28.4, 26.8, 23.9, 22.8, 18.8, 0.0. HRMS (ESI): *m/z* $[M+H]^+$calcd for $C_{24}H_{32}NO_2Si$: 394.2197, found: 394.2192.

^{1}H, and ^{13}C NMR spectra of 2*H*-pyran compound 1 compounds 3a-3j

$^{13}C\{^{1}H\}$ NMR (100 MHz, $CDCl_3$) spectrum of compound 3a

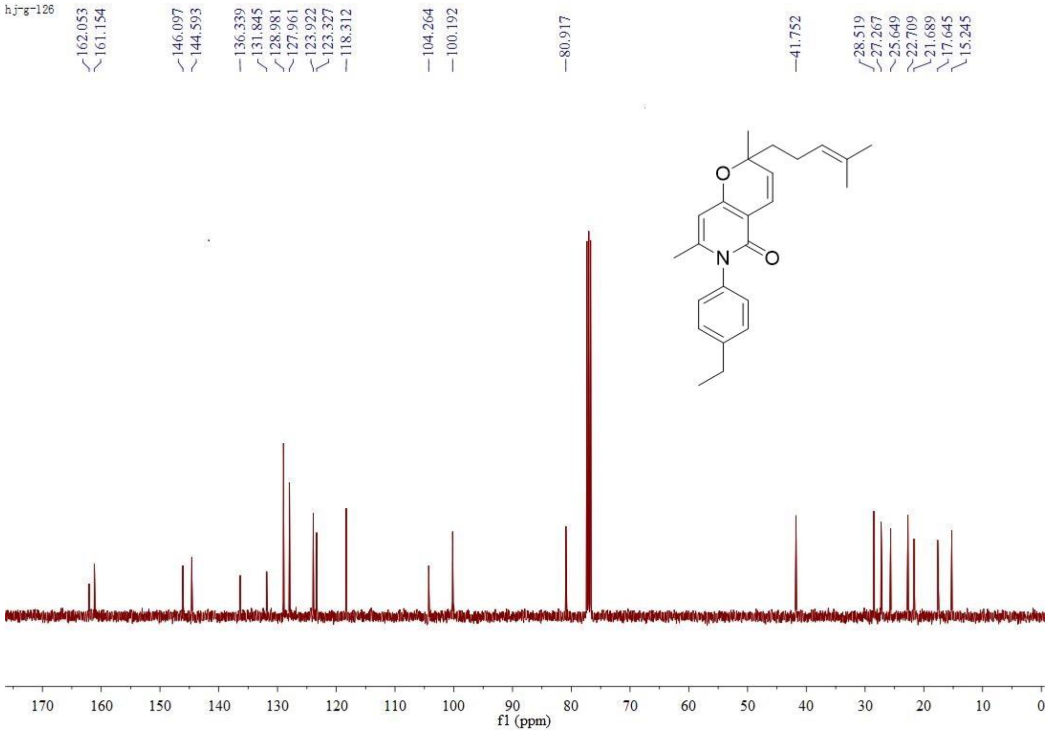

^{1}H NMR (400 MHz, $CDCl_3$) spectrum of compound 3a (eluent: PE/EA = 5:1)

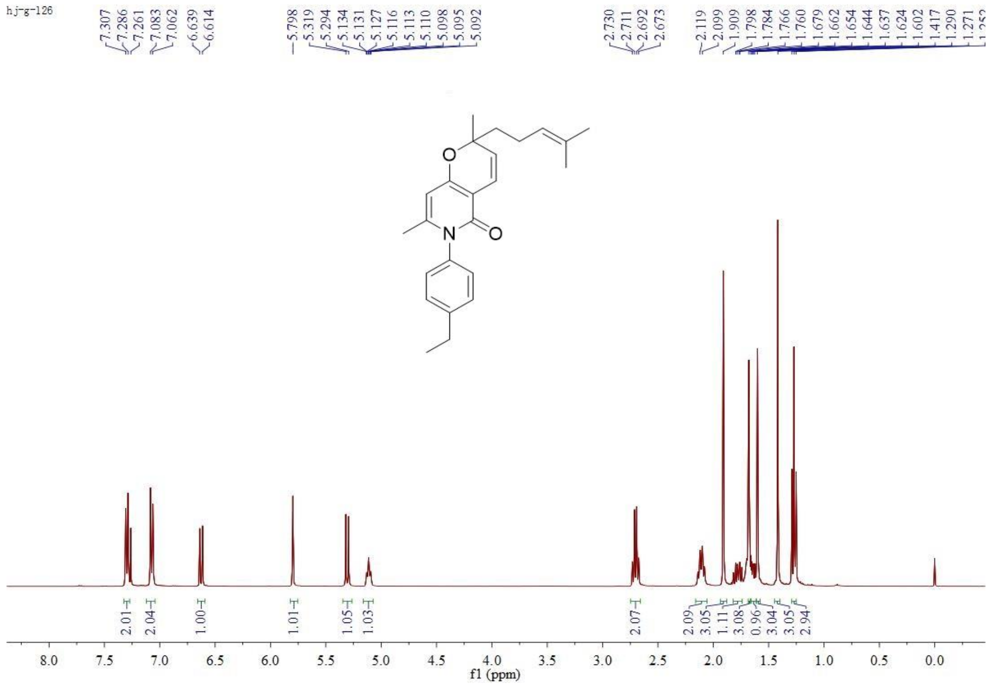

^{13}C{^{1}H} NMR (100 MHz, $CDCl_3$) spectrum of compound 3b

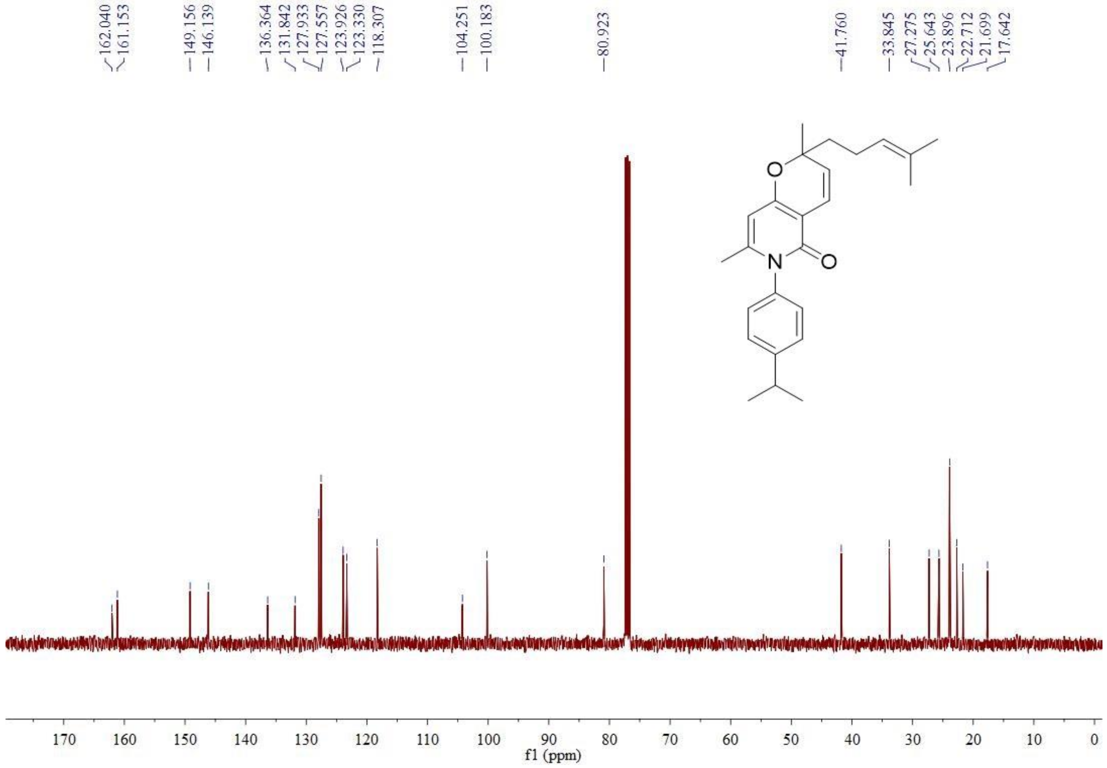

^{1}H NMR (400 MHz, $CDCl_3$) spectrum of compound 3b (eluent: PE/EA = 5:1)

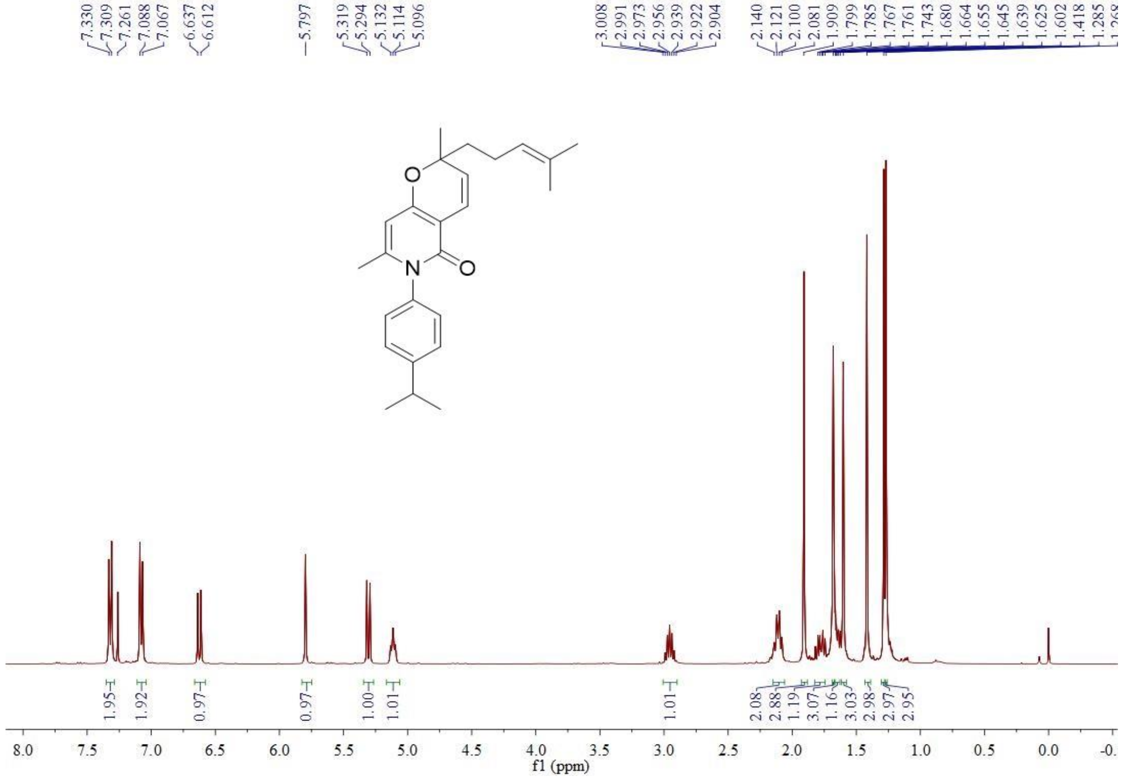

$^{13}C\{^1H\}$ NMR (100 MHz, $CDCl_3$) spectrum of compound 3c

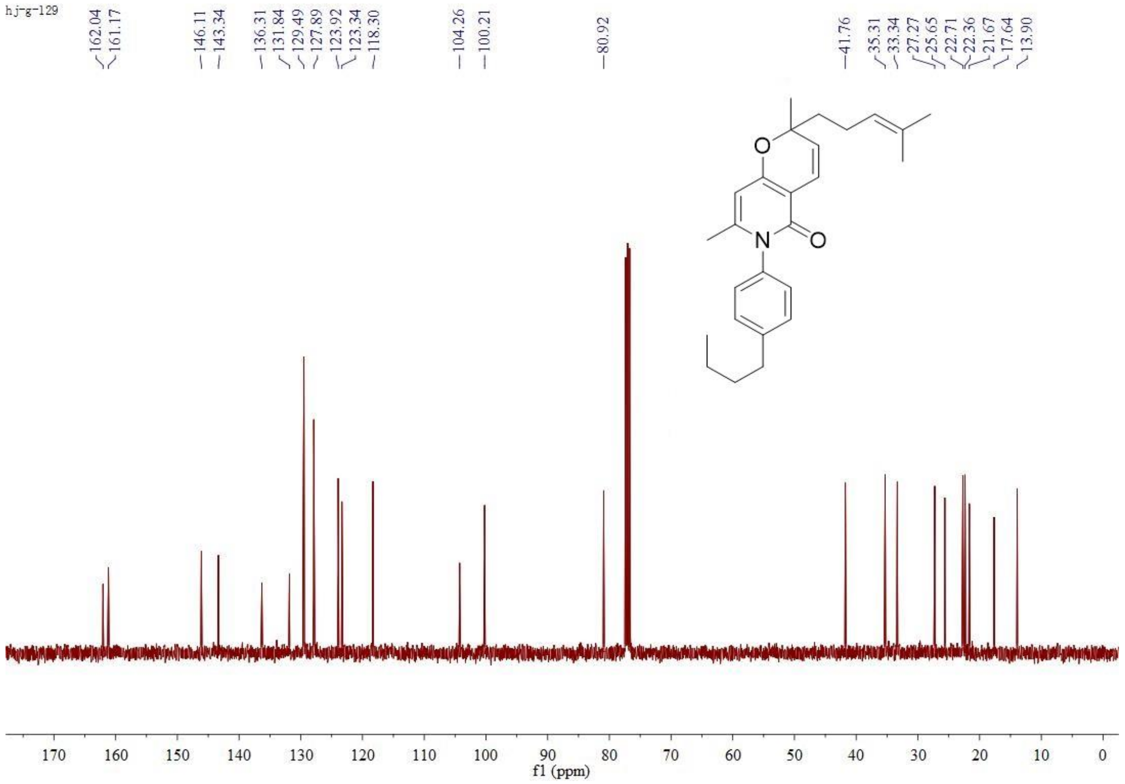

^1H NMR (400 MHz, $CDCl_3$) spectrum of compound 3c (eluent: PE/EA = 5:1)

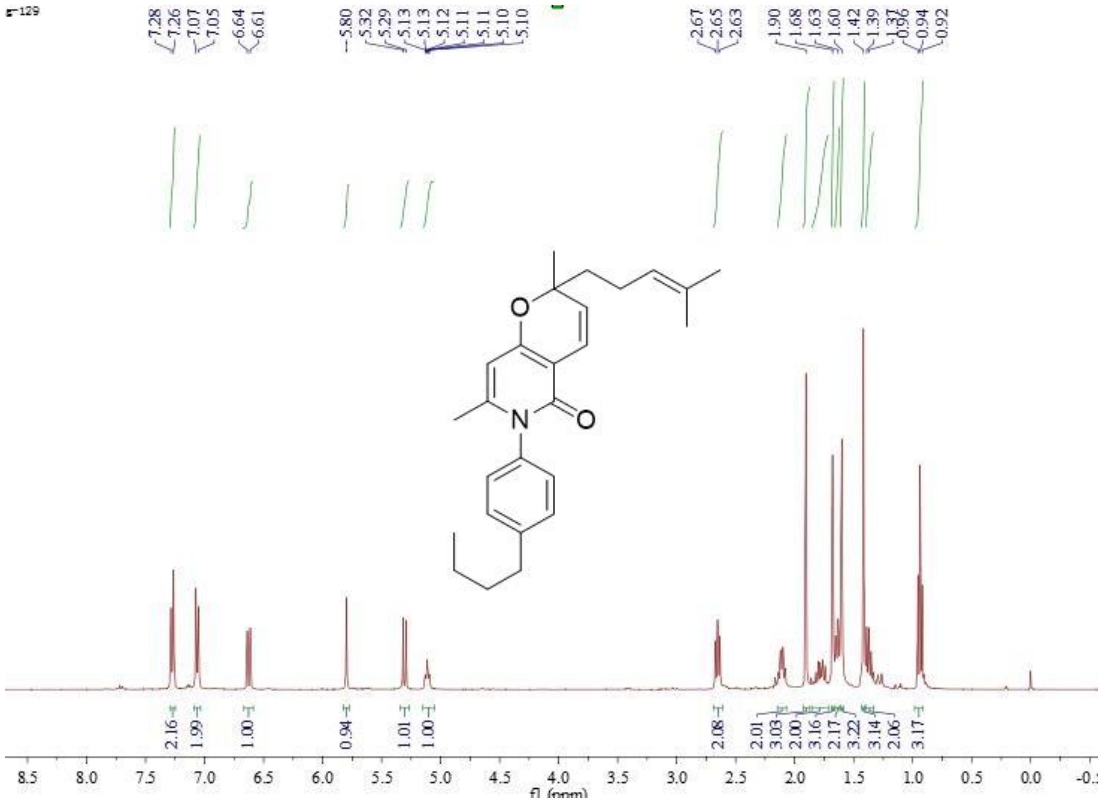

$^{13}C\{^{1}H\}$ NMR (100 MHz, $CDCl_3$) spectrum of compound 3d

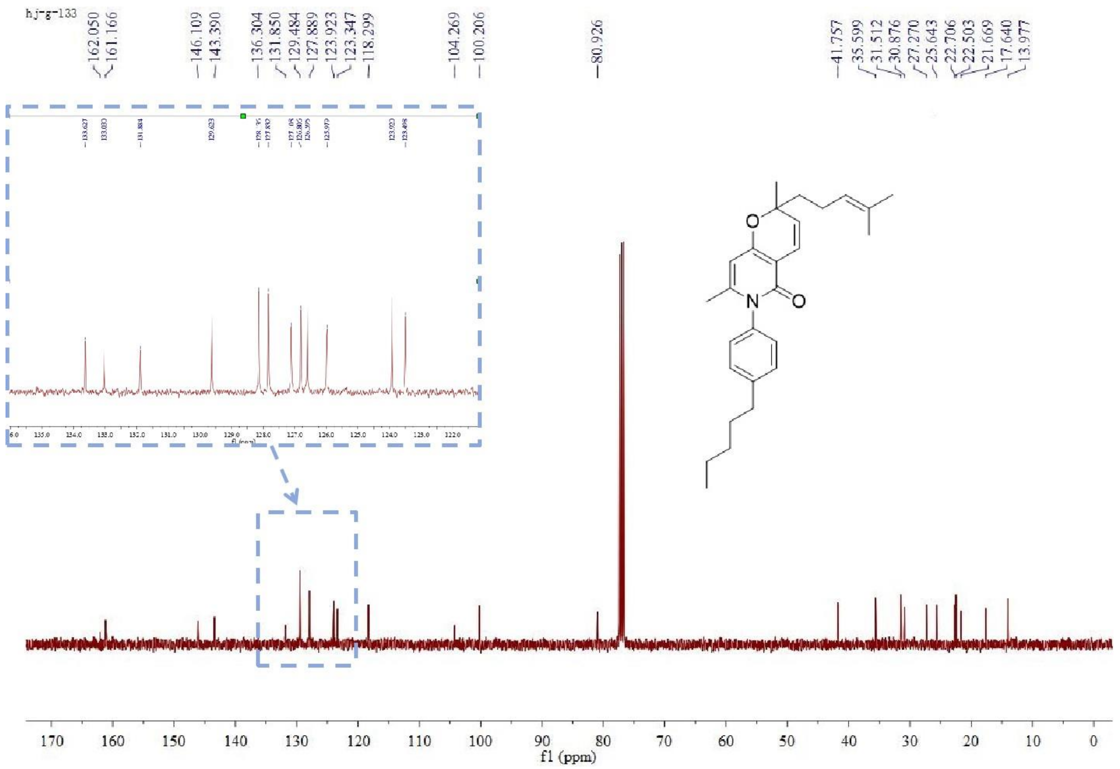

^{1}H NMR (400 MHz, $CDCl_3$) spectrum of compound 3d (eluent: PE/EA = 5:1)

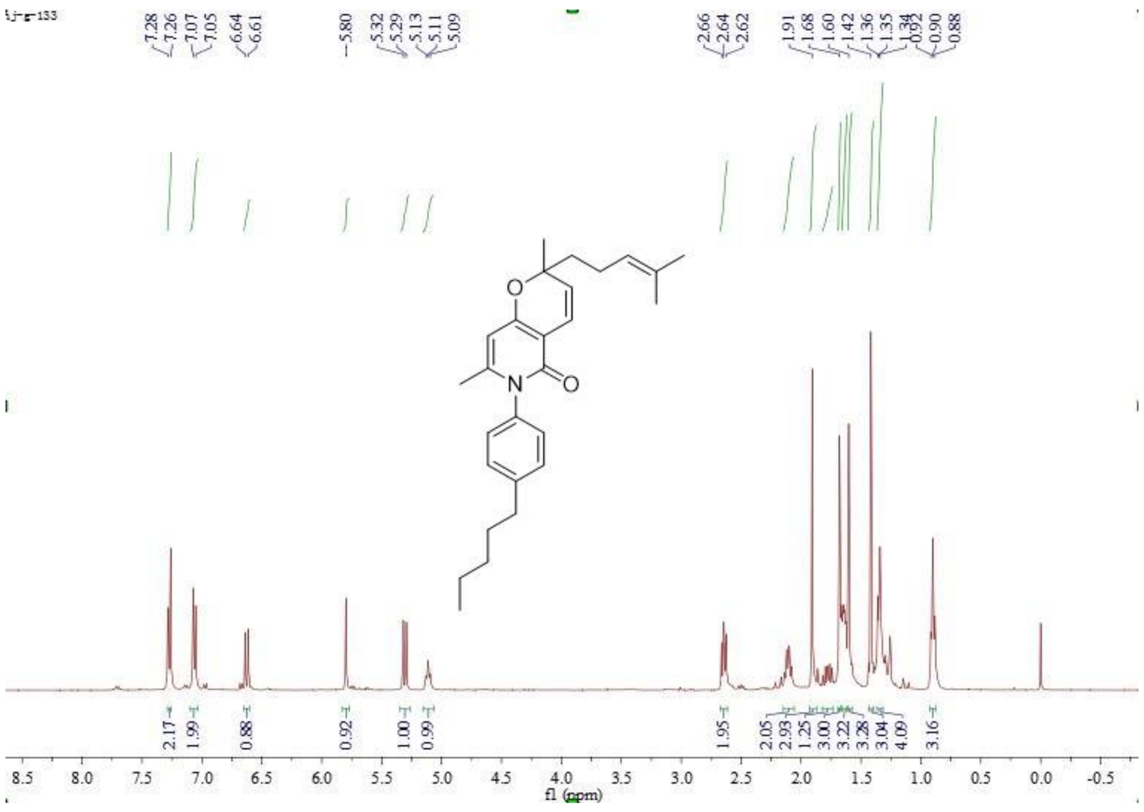

$^{13}C\{^{1}H\}$ NMR (100 MHz, $CDCl_3$) spectrum of compound 3e

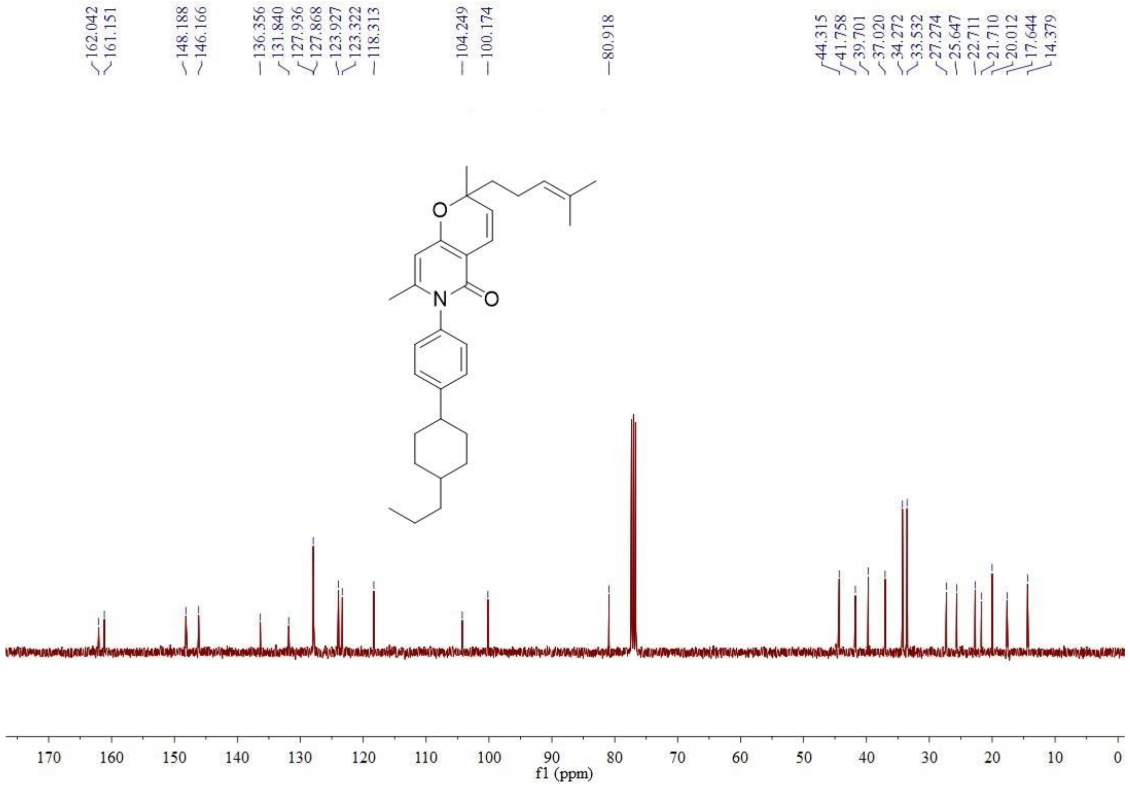

^{1}H NMR (400 MHz, $CDCl_3$) spectrum of compound 3e (eluent:

PE/EA = 5:1)

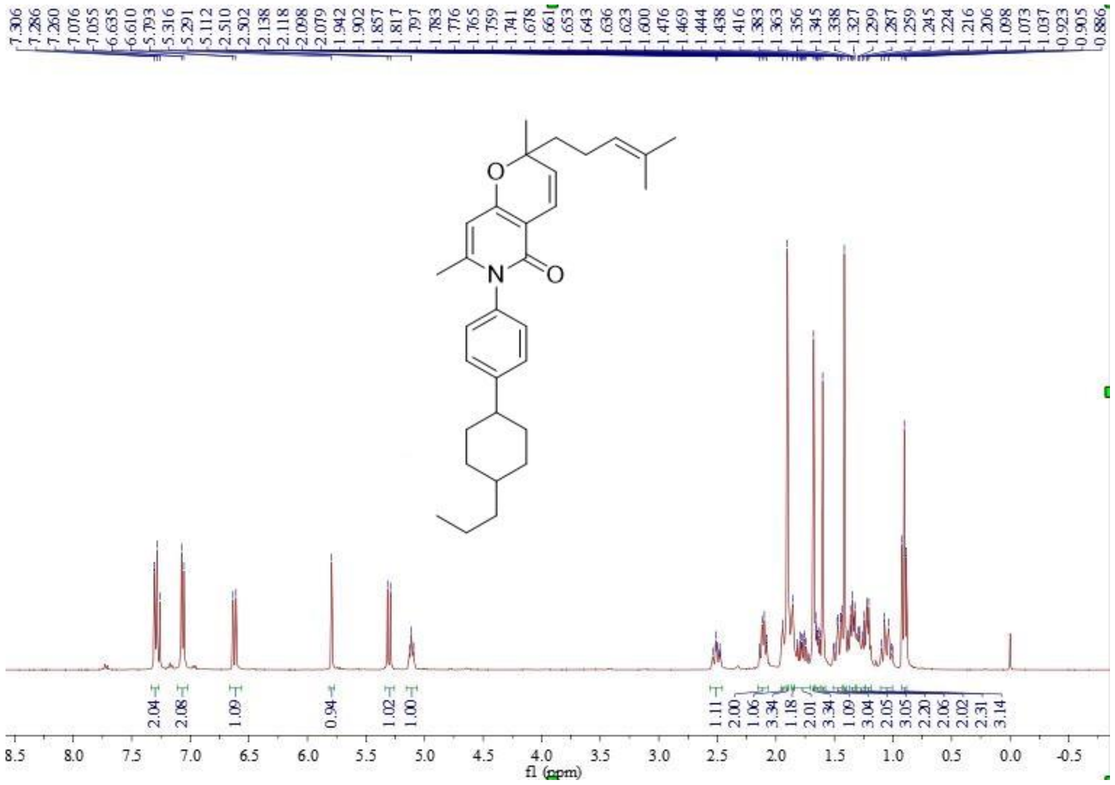

$^{13}C\{^1H\}$ NMR (100 MHz, $CDCl_3$) spectrum of compound 3f

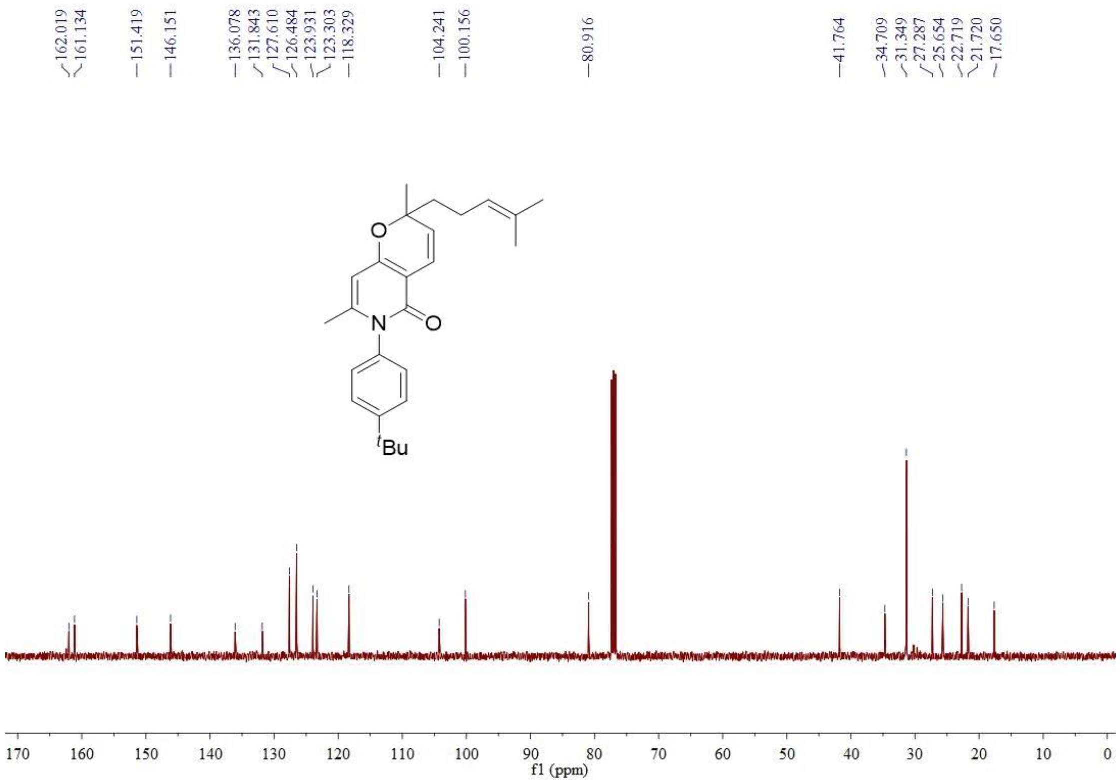

^1H NMR (400 MHz, $CDCl_3$) spectrum of compound 3f (eluent: PE/EA = 5:1)

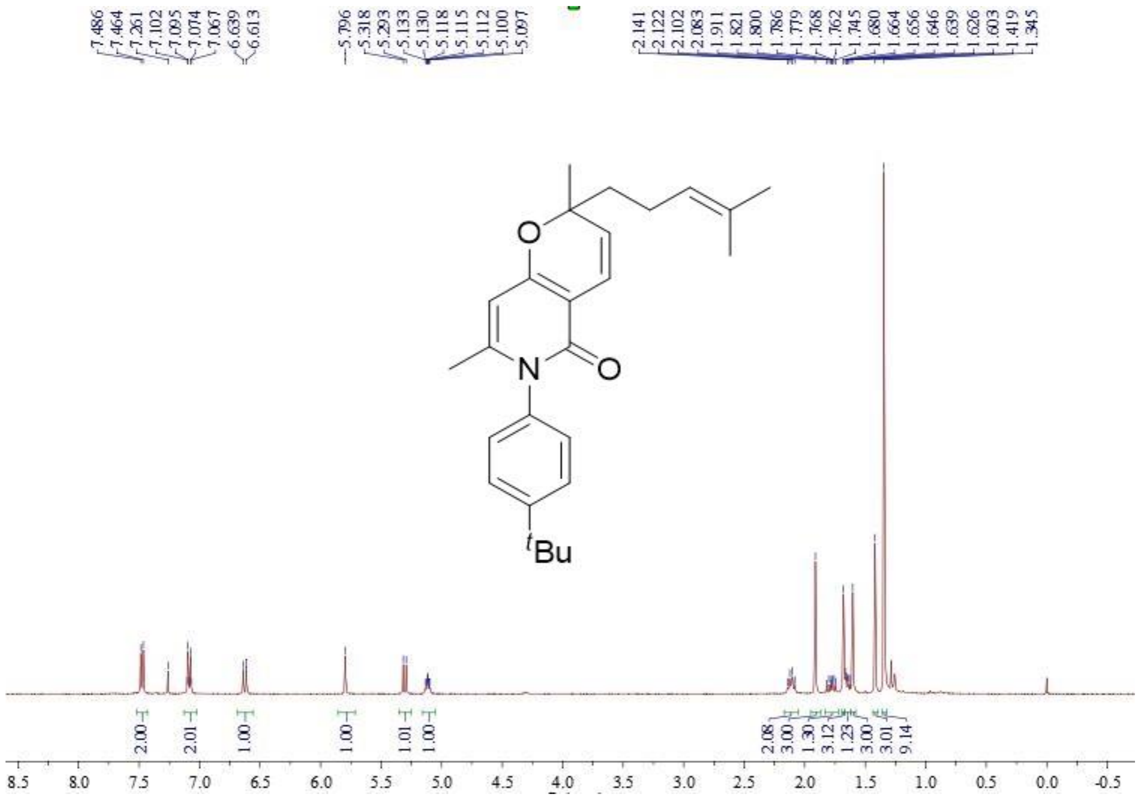

$^{13}C\{^{1}H\}$ NMR (100 MHz, $CDCl_3$) spectrum of compound 3g

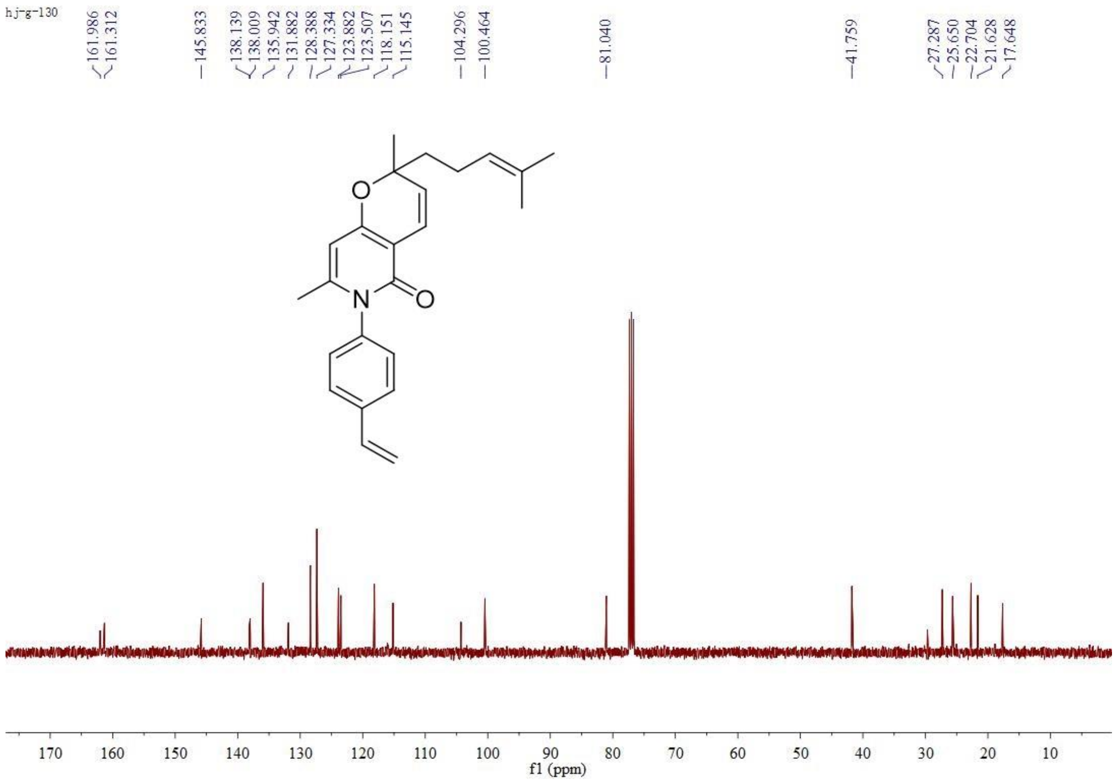

^{1}H NMR (400 MHz, $CDCl_3$) spectrum of compound 3g (eluent:

PE/EA = 5:1)

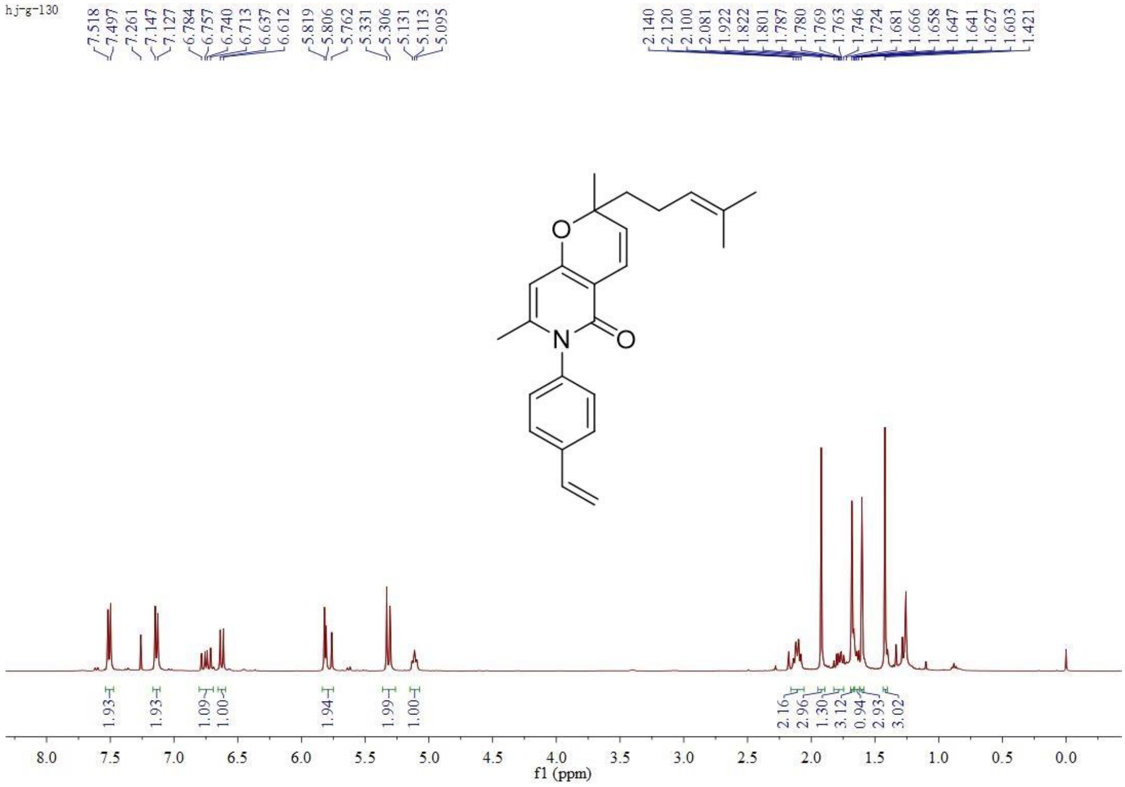

$^{13}C\{^{1}H\}$ NMR (100 MHz, $CDCl_3$) spectrum of compound 3h

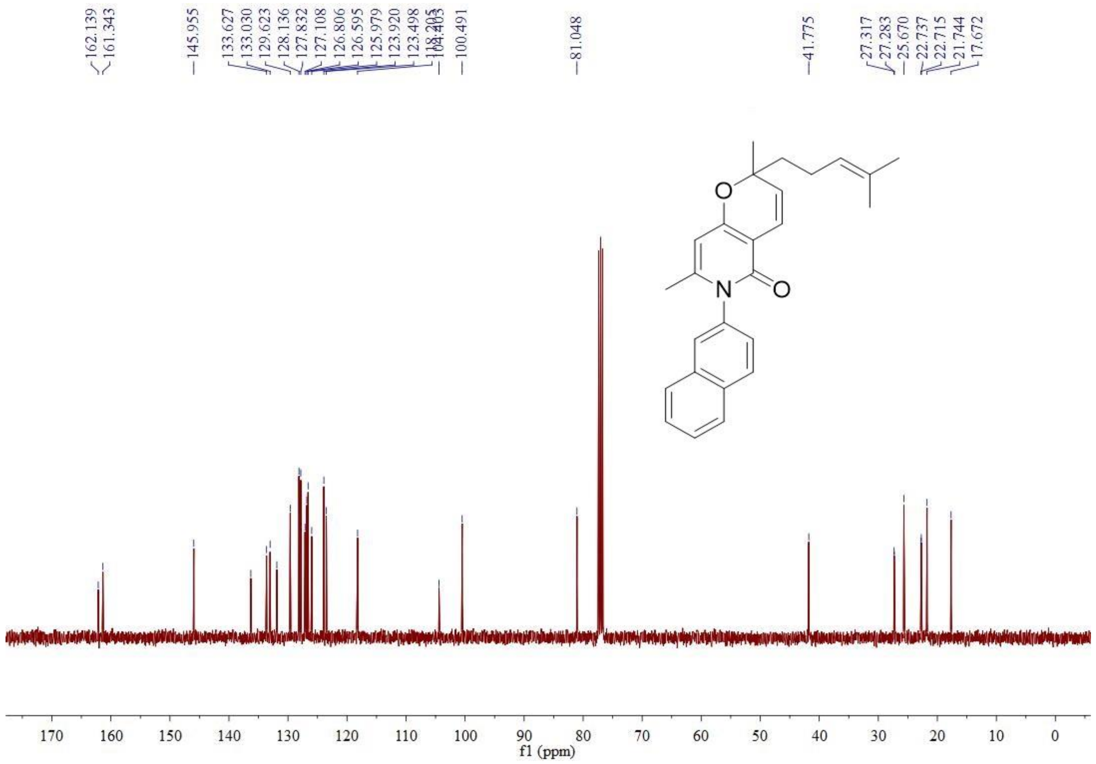

^{1}H NMR (400 MHz, $CDCl_3$) spectrum of compound 3h (eluent: PE/EA = 5:1)

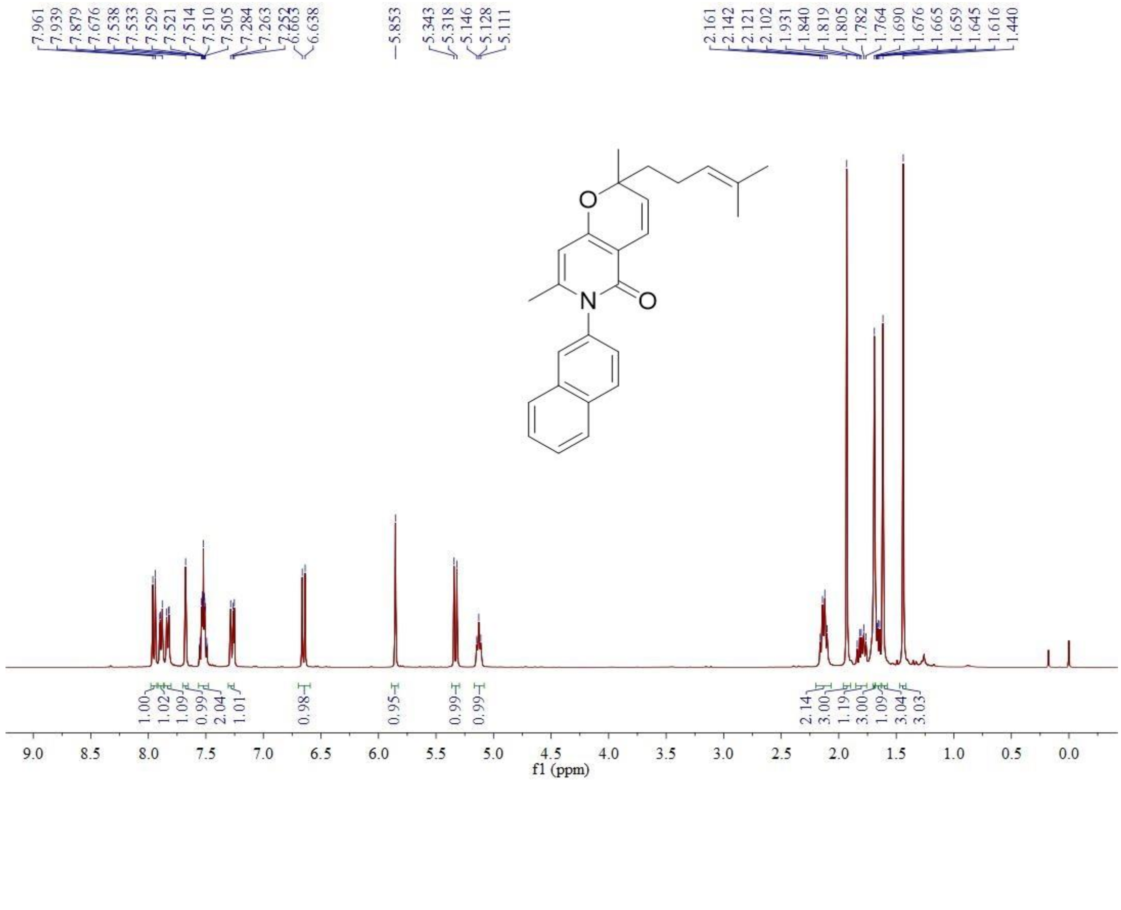

$^{13}C\{^{1}H\}$ NMR (100 MHz, $CDCl_3$) spectrum of compound 3i

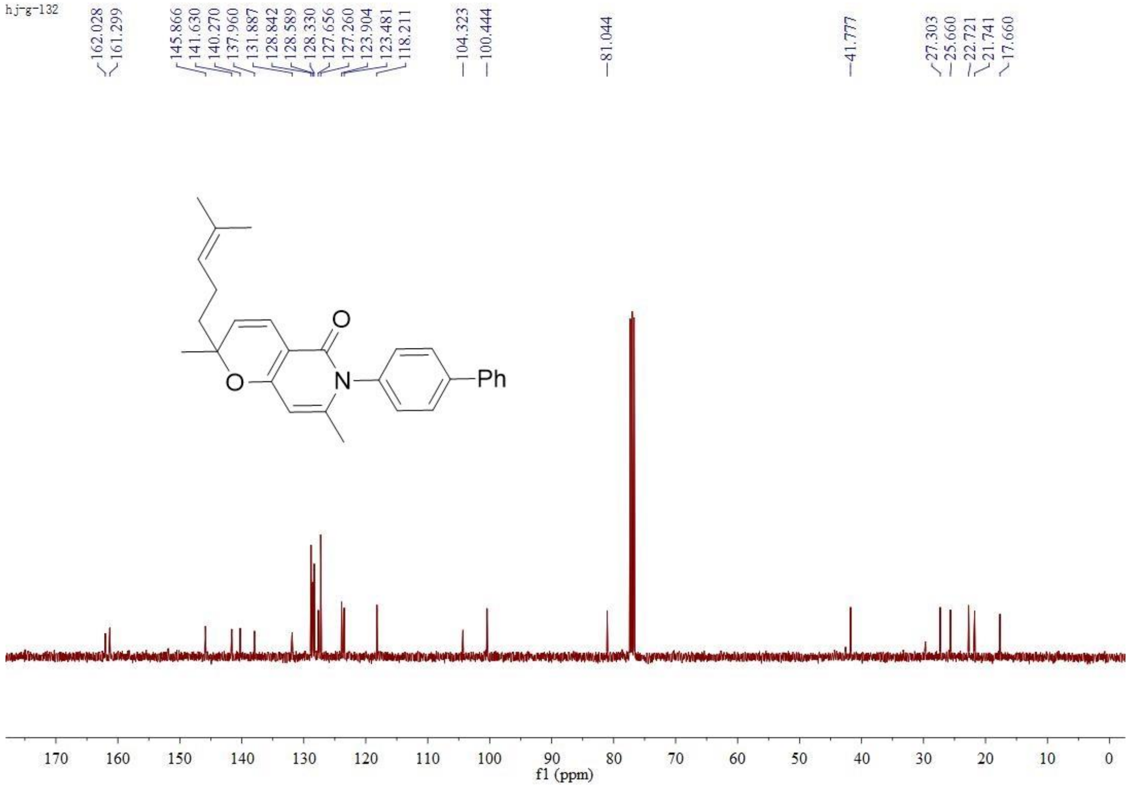

^{1}H NMR (400 MHz, $CDCl_3$) spectrum of compound 3i (eluent: PE/EA = 5:1)

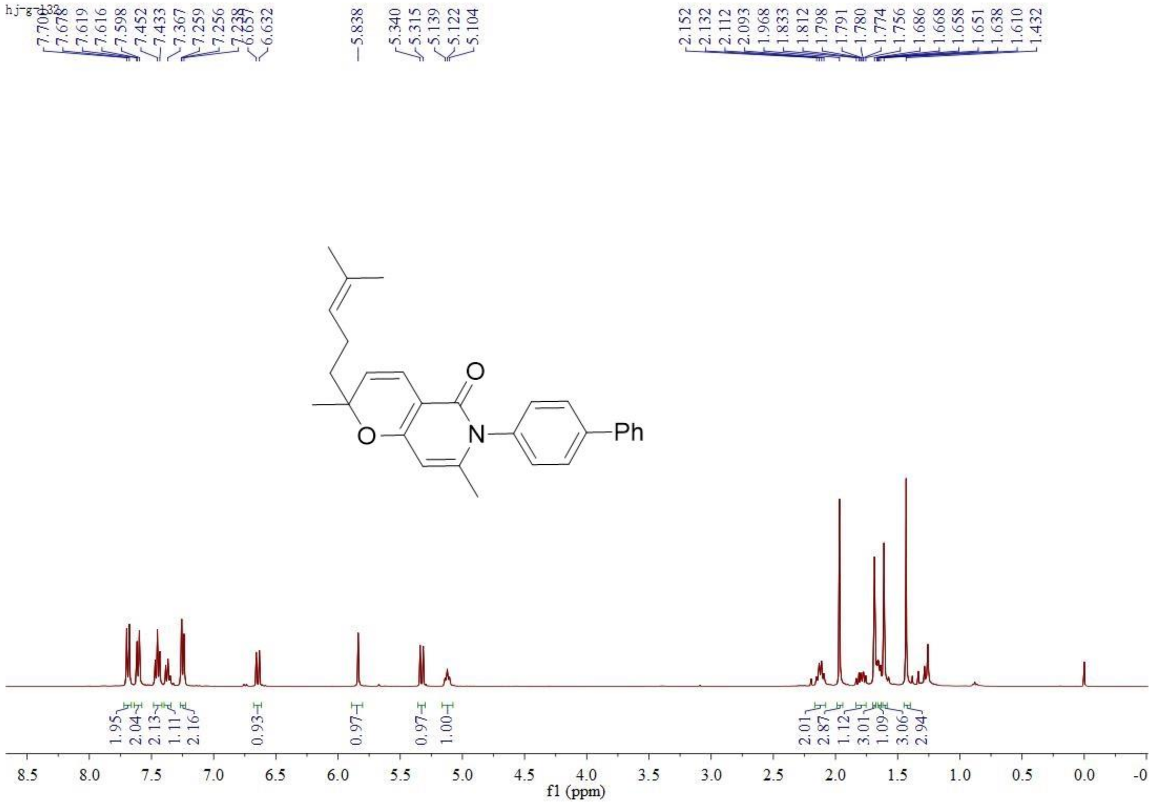

$^{13}C\{^1H\}$ NMR (100 MHz, $CDCl_3$) spectrum of compound 3j

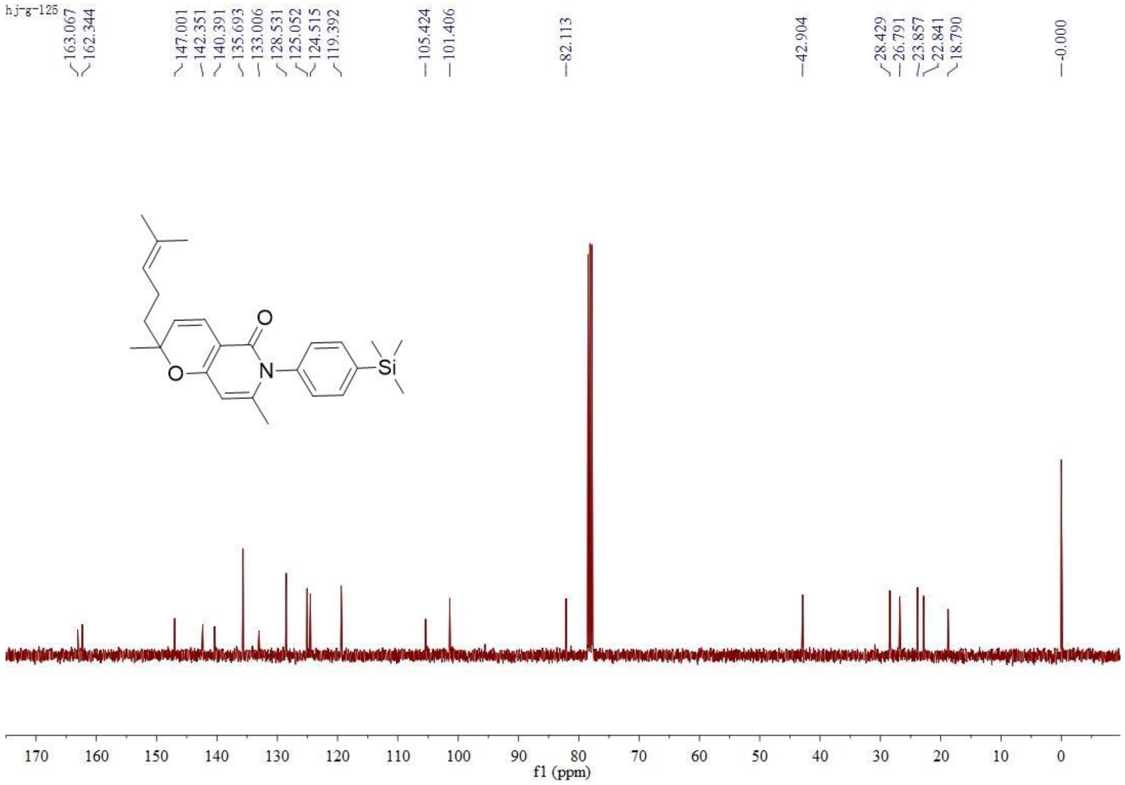

^1H NMR (400 MHz, $CDCl_3$) spectrum of compound 3j (eluent: PE/EA = 5:1)

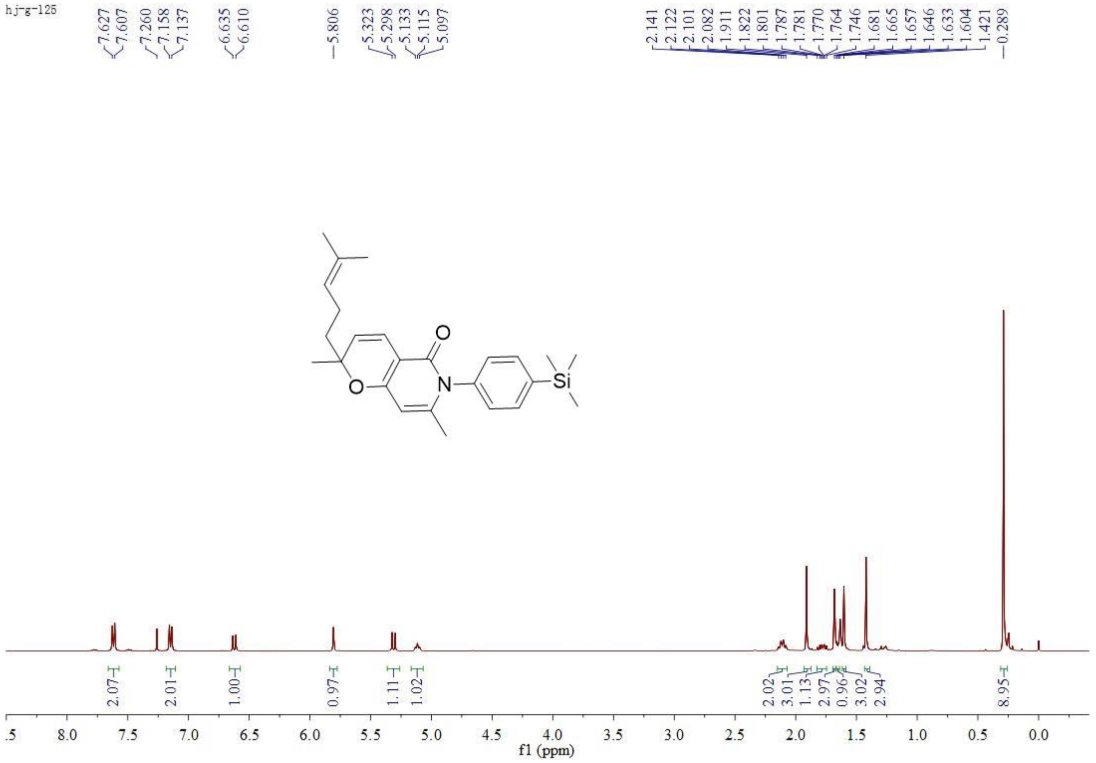

For the quantitative real-time PCR primers.

Gene	Forward（5’-3’）	Reverse（5’-3’）	PCR length（bp）
BCL-2	TGGTGGAGGAGCTCTTCAGG	ATCCTGGATCCAGGTGTGCA	177
BAX	ATCAGAACCATCATGGGCTG	TCTTCCAGATGGTGAGTGAG	222
Caspase3	TCGGTCTGGTACAGATGTCG	CTTCACCATGGCTCAGAAGC	182
cytochrome C	GACTCCTGACCTCGTGATCC	ATCTGTGCCAACACAGACCT	128
GADPH	GAAGGTGAAGGTCGGAGTC	GAAGATGGTGATGGGATTTC	226

HPLC spectra of compound **3h**

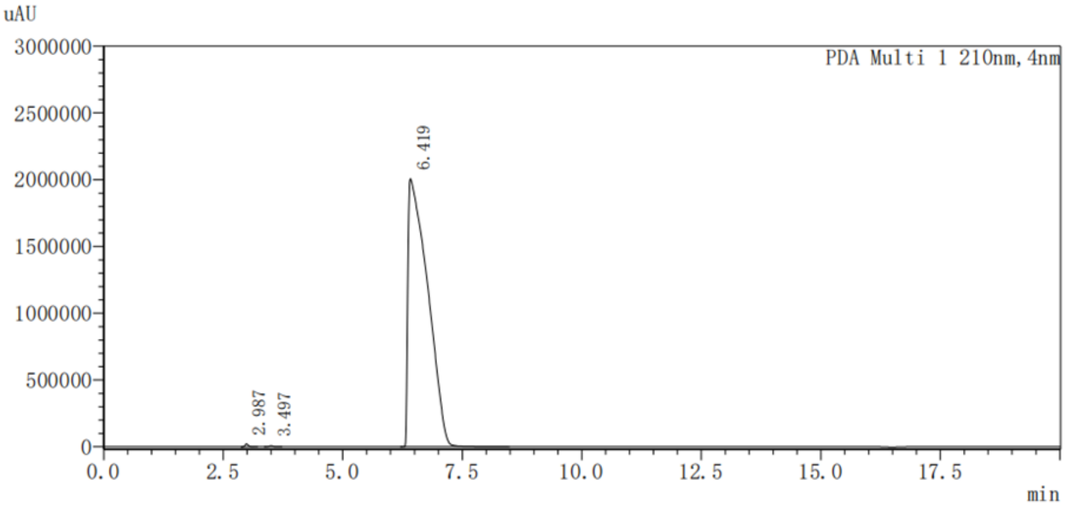

PDA Ch1 210nm

Peak number	Retention time	Area	Height	Concentration
1	2.987	104649	21544	0.181
2	3.497	41597	7260	0.072
3	6.419	57691399	2006361	99.747
Total		57837645	2035165	
